# Syntheses, characterization, and biological activity of novel mono- and binuclear transition metal complexes with a hydrazone Schiff base derived from a coumarin derivative and oxalyldihydrazine

**DOI:** 10.1007/s00706-017-2075-9

**Published:** 2017-11-27

**Authors:** Esther Theresa Knittl, Azza A. Abou-Hussein, Wolfgang Linert

**Affiliations:** 1 Institute of Applied Synthetic Chemistry, Vienna University of Technology, Getreidemarkt, 9/163-AC, 1060 Vienna, Austria; 2 Faculty of Women for Arts, Science and Education, Ain Shams University, Heliopolis, Cairo, Egypt

**Keywords:** 3-Formyl-4-hydroxycoumarin derivative, Mono- and binuclear complexes, Cyclic voltammetry, Antibacterial and antifungal activity

## Abstract

**Abstract:**

A hydrazone Schiff base ligand was synthesized by the condensation of 3-formyl-4-hydroxycoumarin and oxalyldihydrazide in the molar ratio 2:1. The Schiff base ligand acts as a mono-, bi-, tri- or even tetradentate ligand with metal cations in the molar ratios 1:1 or 2:1 (*M*:*L*) to yield either mono- or binuclear complexes as keto or enol isomers, where *M* = Co(II), Ni(II), Cu(II), VO(IV), and Fe(III). The ligand and its metal complexes were characterized by elemental analyses, IR, ^1^H NMR, mass, and UV–Vis spectroscopy. Furthermore, the magnetic moments were calculated from the measured electric conductivities of the complexes. According to the received data, the dihydrazone ligand contains one or two units of ONO domains and can bind to the metal ions via the azomethine nitrogen, the carbonyl oxygen atoms, and/or the phenolic oxygen atoms. Electronic spectra and the magnetic moments of all complexes show that the complexes’ geometries are either octahedral, tetrahedral, square planar, or square pyramidal. Cyclic voltammograms of the mononuclear Co(II) and Ni(II) complexes show quasi-reversible peaks. Tests against two pathogenic bacteria as Gram-positive and Gram-negative bacteria for both, the Schiff base ligand and its metal complexes were carried out. In addition, also one kind of fungi was tested. The synthesized complexes demonstrate mild antibacterial and antifungal activities against these organisms.

**Graphical abstract:**

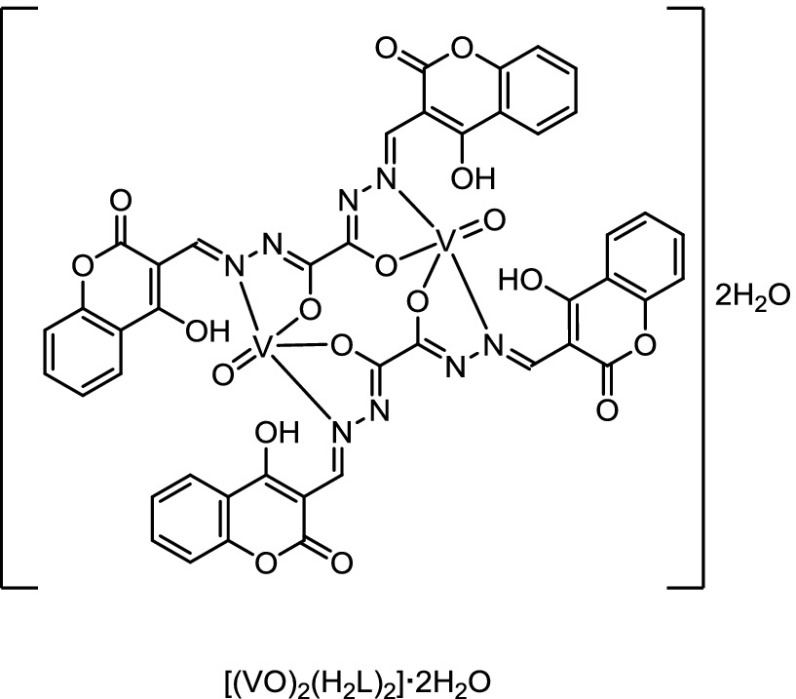

**Electronic supplementary material:**

The online version of this article (doi:10.1007/s00706-017-2075-9) contains supplementary material, which is available to authorized users.

## Introduction

The chemistry of organic hydrazone compounds, which include –N–NH–CO– groups, takes the forefront position in the development of coordination chemistry of symmetrical dihydrazone transition metal complexes, as they demonstrate versatility in their coordination, a tendency to show stereochemistry [1–5] due to higher coordination numbers, an ability to act in their neutral or deprotonated forms, as they behave as keto–enol tautomers, bearing unusual coordination numbers [2, 6–9] and flexibility in assuming different conformations. They are extensively used due to their promising applications in biomimetic catalytic reactions, analytical chemistry [10–12], polymer coating pigments, industrial fluorescent materials, Portland cement, amphibolites, and granites [13]. They bear remarkable antifungal, antibacterial, anticancer, antiviral, and herbicidal applications [14–18] and anti-inflammatory properties, exhibited by these compounds, which can be attributed to their metal complexing abilities [19–21]. Based on their applicability in various fields, we are extending this field by the synthesis of novel mono- and binuclear hydrazone complexes.

The present study is an extension to our research work for the formation of mono- and binuclear complexes for coumarin derivatives, synthesized by the condensation of 4-hydroxycoumarin with oxalyldihydrazide in the molar ratio of 1:2, to afford the corresponding hydrazine [22]. Mono- and binuclear complexes of Co(II), Ni(II), Cu(II), VO(IV), and Fe(III) with the hydrazone Schiff base ligand have been prepared. H_4_L acts as a bidentate, tridentate, tetradentate, or hexadentate ligand in a mono- or binuclear form. The bonding sites are the azomethine nitrogen, the carbonyl oxygen in its keto or enol isomeric form, and the phenolic oxygen. The ligand exhibits a tautomerization, which can modulate the coordination to the metal in the keto or enol form. The redox properties and the nature of the electro-active species of the complexes have been characterized to study the electrochemical behavior of selected complexes by cyclic voltammetry [23]. It was hoped that the bacterial activity of the present hard-soft Schiff base ligand and its transition metal complexes show increased activity against two pathogenic bacteria and fungi. As Gram-positive bacteria, Staphylococcus aureus was used, and as Gram-negative bacteria, Pseudomonas fluorescens was taken. To investigate anti-fungi, Fusarium oxysporum was used as an example.

## Results and discussion

The data on the characterization of the synthesized ligand and its transition metal complexes are reported in Table 1.

**Table 1:** Data on the characterization of the synthesized ligand and its transition metal complexes

Complex/ligand		Electronic absorption bands nm^−1^ and assignment		*µ*_eff_	*Λ* ^a^	Geometry	*M*/g mol^−1^	Yield/%	Color
		*d–d* transition	Assignment						
**1**	[Co(H_3_L)(NO_3_)]·2H_2_O	610 (0.074), 675 (0.054)	^4^A_2_(F) → ^4^T_1_(P)	4.22	95	Tetrahedral	618.33	56	Brownish blue
**2**	[Ni(H_3_L)(H_2_O)]NO_3_	652 (0.045), 766 (0.052)	^3^A_2_(F)(*ν*_3_) → ^3^T_1g_(P)	2.92	91	Tetrahedral	600.08	51	Brownish red
**3**	[Cu(H_2_L)]·2H_2_O	439 (0.330), 567 (0.424)	^2^B_1g_ → ^2^A_1g_, ^2^B_1g_ → ^2^E_g_	1.97	–	Square planner	559.93	71	Green
**4**	[(VO)_2_(H_2_L)_2_]·2H_2_O	613 (0.414), 754 (0.378)	^1^B_2_ → ^2^E, ^1^B_2_ → ^2^A_1_	1.81	–	Square pyramidal	1090.63	54	Green
**5**	Fe_2_(H_2_L)_2_(NO_3_)_2_(H_2_O)_2_	553 (0.021), 720 (0.027)	Charge transfer from UV to Vis region	4.35	145	Octahedral	1192.44	70	Black
**6**	Co_2_(H_2_L)(NO_3_)_2_(H_2_O)_2_	575 (0.076), 660 (0.088)	^4^A_2g_(F) → ^4^T_1g_ (F)(*ν*_2_) ^4^A_2g_(P) → ^4^T_1g_ (P) (*ν*_3_)	4.69	153	Octahedral	738.62	61	Brown
**7**	Ni_2_(H_2_L)(NO_3_)_2_(H_2_O)_4_	652 (0.053), 435 (0.076)	^3^A_2g_(F) → ^3^T_1g_ (F)(*ν*_2_) ^3^A_2g_(F) → ^3^T_1g_ (P) (*ν*_3_)	3.29	158	Octahedral	773.81	56	Green
**8**	[Cu_2_(H_2_L)(H_2_O)_4_](NO_3_)_2_	610 (0.432), 455 (0.339)	^2^A_1g_ → ^2^B_1g_, ^2^E → ^2^B_1g_	1.83	154	Square pyramidal	783.52	62	Violet
**9**	[(VO)_2_(H_2_L)(SO_4_)]·3H_2_O	624 (0.532), 775 (0.388)	^1^B_2_ → ^2^E, ^1^B_2_ → ^2^A_1_	1.74	–	Square pyramidal	744.34	71	Green
**10**	Fe_2_(L)(H_2_O)_4_(NO_3_)_2_	520 (0.034), 765 (0.044)	Charge transfer from UV to Vis region	4.87	162	Octahedral	766.10	53	Black
	H_4_L	–	–	–	–	–	462.37	71	Yellow

^a^ Molar conductivities in Ohm^−1^ cm^2^ mol^−1^

### Characterization of the Schiff base H_4_L

H_4_L was analyzed by elemental analysis, IR, UV–Vis, mass spectrometry, and NMR spectroscopy. The mass spectrum and the schematic fragmentation of the ligand can be seen in the supplementary materials S1.

The IR spectrum is consistent with the structure of H_4_L. In fact, the ligand includes four acidic hydrogen atoms, which can be released easily by adding a base. Thus, it can act in its mono-, di-, tri- or even tetra-deprotonated form, when binding to a metal center. Due to keto–enol tautomerism, it can exist in both forms, or in a mixture of both, since there is an amide group (–NH–C=O) in the ligand structure. However, the IR spectrum indicates that the ligand exists almost in its keto form in the solid state. The bands, which appeared at 1695 *ν*(C=O), 3142–3180 *ν*(NH) + 1505 *ν*(C–N) + *δ*(N–H), 1215 *δ*(N–H), 743 cm^−1^ *Φ*(C=O) are characteristic for the amide group and thus support the existence of the tautomeric keto form of the ligand [24, 25]. It is worth to mention that the IR spectrum of the ligand displays another band at 1723 cm^−1^, which can be attributed to the presence of the lactone group in the coumarin ring [28, 29]. The band observed at 1272 cm^−1^ is assigned to *ν*(C–O–C) of the coumarin ring [30], while the band at 1253 cm^−1^ is ascribed to the phenolic C–O stretching vibrations [31], and another one in the region from 1567 to 1483 cm^−1^ is assigned to the combination of *ν*(C=C) of the aromatic ring system [32]. The broad band in the region of 3142–3180 cm^−1^ in the free ligand is assigned to the stretching vibration of the amide group (NH).

^1^H and ^13^C NMR spectra are given in the supplementary material S2. In the ^1^H NMR spectrum of the ligand, a very small signal could be observed at 13.36 ppm, which may be attributed to the tautomerism of the ligand [26, 27]. The broad signal at 12.43 ppm, *H*(e,e′), corresponds to the hydroxyl groups. This signal disappears after the addition of D_2_O, which indicates that these protons are acidic.

### Characterization of the complexes of H_4_L: IR spectroscopy

Infrared spectra of the complexes were recorded to gain information about the binding modes of the ligand to the corresponding metals. All spectra can be seen in the supporting material S3. In fact, it could be observed in the IR spectra that the ligand can be coordinated in its keto or enol form. For the monometallic complexes [Co(H_3_L)(NO_3_)]·2H_2_O (**1**) and [Ni(H_3_L)(H_2_O)]·NO_3_ (**2**), the great flexibility of the ligand and, thus, the presence of numerous donor sites make it difficult to establish the coordination types of the final complexes; however, IR spectra and elemental analysis suggest that at least one oxygen atom from one phenolic site and its imine nitrogen, as well as one terminal carbonyl group, are involved in the coordination. For the complexes **1** and **2,** the strong band ascribed to the vibration of the C=O bond of the hydrazone Schiff base (ca 1695 cm^−1^) appears at the same position in the spectra of the complexes, but with a much lower intensity. In addition, a new band appears at 1685 and 1681 cm^−1^ in the spectra of the complexes **1** and **2**, respectively, which suggests that the oxygen atom on one side of the ligand coordinates to the metal atom, while the other C=O group remains free. In a similar way, there are observed two bands assigned to the stretching frequencies of the azomethine groups at 1624 and 1618 cm^−1^, which could give a hint that the azomethine group on one side of the ligand coordinates to the metal, while the same group on the other side stays uncoordinated [33]. One broad band, which appears in the spectra of the ligand and the corresponding metal complexes of Co(II) and Ni(II) in the range between 3162 and 3156 cm^−1^, is assigned to the stretching vibration of *ν*(N–H). Finally, the IR spectra of the complexes show two bands, which can be associated with the stretching frequencies of the coordinated and uncoordinated phenolic oxygens in the ranges between 1232–1237 and 1260–1267 cm^−1^, respectively. The observations indicate that the Schiff base ligand acts as a mono-negative, tridentate ligand in the case of the synthesized Co(II) and Ni(II) complexes, where one moiety of the Schiff base is coordinated to the metal atoms by only one carbonyl, azomethine, and phenolic oxygen group, forming six- and five-membered chelate rings with the metal ions, forming the complexes **1** and **2** [34]. In fact, this is not the case for [Cu(H_2_L)]·2H_2_O (**3**), where both sides of the ligand participate in the coordination to the metal ion, in spite of the Schiff base being present in its keto form. The carbonyl groups remain uncoordinated in this case [35]. The bands in the IR spectra, assigned to *ν*(N–H) and *ν*(C=O), were found nearly at the same position in the spectrum of the complex and the ligand, indicating that the possibility of the coordination of the ligand to the metal center by these groups is quite low. On the other hand, the azomethine group is shifted to lower frequencies, which support the coordination by the two azomethine groups to the metal ion. This is further consistent with the involvement of the phenolic oxygen atoms, which also bind to the metal center in the final complex. These facts are confirmed by elemental analyses, physical, and spectroscopic measurements.

For the macrocyclic complex [(VO)_2_(H_2_L)_2_]·2H_2_O (**4**), the stretching vibrations of the C=O, as well as the N–H bands, are absent in the spectrum of the complex. This could be attributed to the enolization of both carbonyl groups of the Schiff base during the formation of the complex [36]. The appearance of a new C–O band in the spectrum of the complex at 1257 cm^−1^ suggests that the ligand coordinates by the two C–O groups of the oxalylhydrazine moiety to the metal ion after the deprotonation of this group [37]. The band assigned to C=N has been shifted to lower frequencies in the spectrum of the complex compared to the one of the free ligand, which suggests that both azomethine nitrogens participate in the coordination. Also, the spectrum of the complex shows an additional C=N band at 1616 cm^−1^, which might be assigned to the formation of two new C–N groups as a matter of the tautomeric enolization [38]. Due to the fact that the stretching (*ν* 3355 cm^−1^) and bending (*δ* 1360 cm^−1^) vibrations of the hydroxyl groups of the coumarin rings did not change their positions in both spectra, it indicates that these groups do not take part in the coordination. In the case of Fe_2_(H_2_L)_2_(NO_3_)_2_(H_2_O)_2_ (**5**), the stretching vibration assigned to the carbonyl group of the hydrazone Schiff base shows no significant changes, when the spectrum of the complex is compared to the one of the free Schiff base due to the presence of one free C=O group, which is not involved in the coordination [39]. However, a new band appeared in the spectrum of the complex between 855 and 842 cm^−1^, which might be attributed to the participation of the C–O group in the complexation, which results in the formation of the enol form of the amide groups (–N=C–OH). Another evidence for the enolization is the presence of the stretching frequencies of the azomethine groups C=N in the spectrum of the metal complex. In fact, the *ν*(–C=N) bands were found in two different regions. Before, it was observed at 1623 cm^−1^, which is lower than the *ν*(–C=N) of the free ligand at 1635 cm^−1^ as a result of the coordination to the metal center. The band at 1270 cm^−1^, which is ascribed to the phenolic C–O stretching vibration in the ligand, is shifted to higher frequencies as a result of the coordination to the metal ion and thus, was found at 1282 cm^−1^. It is obvious that the Schiff base behaves as a twofold negative, tridentate ligand in the complex **5**. The coordination occurs via the phenolic oxygen, the azomethine group, and the enolic oxygen.

The IR spectra show that the hydrazone Schiff base ligand behaves in its keto form in the binuclear complexes Co_2_(H_2_L)(NO_3_)_2_(H_2_O)_2_ (**6**), Ni_2_(H_2_L)(NO_3_)_2_(H_2_O)_4_ (**7**), [Cu_2_(H_2_L)(H_2_O)_4_](NO_3_)_2_ (**8**), and [(VO)_2_(H_2_L)(SO_4_)]·3H_2_O (**9**), where the stretching frequency *ν*(NH) appears at the same range in the spectrum of the ligand H_4_L, compared to the ones of the corresponding metal complexes, which proves that this group is not participating in the coordination. The frequency of the carbonyl oxygen in the spectra is shifted to lower frequencies due to the coordination of this atom to the metal ion in the range between 1675 and 1680 cm^−1^, compared to the free ligand at 1695 cm^−1^. A shift in the same way can also be observed for the azomethine group, which indicates that the nitrogens of these groups participate in the coordination [40]. Moreover, the band at 1000 cm^−1^, which is attributed to the vibration *ν*(N–N) of the ligand, is shifted to higher wavenumbers (by ca. 10 cm^−1^) after the complexation [41, 42]. The complexation through the phenolic oxygen atoms, which takes place after the deprotonation of this group, can be proved by the appearance of a band assigned to *ν*(C–O) at much higher frequencies (1274–1266 cm^−1^) in the spectra of all complexes, compared to the spectrum of the free ligand H_4_L (1253 cm^−1^). The shift to higher wavenumbers is expected due to the increase of the double bond character through the resonance in the chelate ring of the free Schiff base [43, 44]. This observation has been further supported by elemental analysis and molar conductivity values, which indicate that there is a substantial dissociation of the complexes in dimethyl formamide (DMF) and supports the coordination of the ligand to the metal ion in its deprotonated dianionic form. On the other hand, the Schiff base ligand coordinates in its enol form in the case of the binuclear complexes **4** and **5**.

One of the most important features of the IR spectra of some complexes is the possibility to prove the coordination behavior of nitrate and sulfate anions. Strong absorption bands are displayed by the IR spectra of the complexes, which are consistent with monodentate, bidentate or ionic nitrate vibrations. In the complexes **5** and **6**, the nitrate anions behave in a coordinated bidentate nature, where it possesses three non-degenerated vibrational modes (ns, *ν*s’, and *ν*as) in the ranges 1443–1468, 1266–1287, and 1033–1021 cm^−1^, respectively. The distance of about 200 cm^−1^ between the vibrations ns and *ν*s’ confirms the bidentate nature of the nitrate group. On the other hand, for the complexes **1**, **7**, and **10**, the nitrate ions are bound to the metals in a monodentate way, corresponding to the C2v symmetry with three non-degenerated modes of vibrations (*ν*s, *ν*s’ and *ν*as), which appear in the ranges of 1356–1372, 1245–1265, and 836–856 cm^−1^, respectively. For the complexes **2** and **8,** a strong band around 1380 cm^−1^ could be observed and assigned to the stretching mode of ionic nitrate [45]. The presence of nitrate in this complex is shown by IR spectroscopy and elemental analysis. The oxovanadium complexes **4** and **9** exhibit a strong band around 985 and 980 cm^−1^, respectively. This reflects the high π-band order of vanadium to the oxygen link of VO^2+^ [46, 47]. The appearance of new bands at 953 (*v*2), 1166 (*v*5), and 647 (*v*6) cm^−1^ in the VO^2+^ complex **9** is an evidence for the bidentate nature of the SO_4_^2−^ anion This observation has been further supported by conductivity measurements and elemental analysis. It is worth to mention that the broad band, which is observed in the IR spectra of the complexes in the range of 3355–4381 cm^−1^, can be ascribed to the stretching vibrations of *ν*(O–H) of the phenolic group, crystalline or coordinated water molecules associated to the complexes. New bands in the spectra of all complexes, which are absent in the spectrum of the free ligand and are located at 534–584 and 426–488 cm^−1^, can be assigned to *ν*(M–O) and *ν*(M–N), respectively [48]. It is obvious that the binding sites of the ligand to the metal ions are the azomethine nitrogen, and the carbonyl oxygen in either the keto or the enol form and/or the phenolic oxygen atoms. The nitrate, sulfate, and chloride anions, as well as coordinated water molecules, enable the satisfaction of the other coordination sites, to complete the geometry of the complexes. It is of interest that none of the carbonyl lactone groups are involved in the coordination and thus, remain unchanged according to the IR spectra [49, 50]. This observation is further supported by the stretching vibration *ν*(C–O–C), assigned to the coumarin ring, which remains nearly unchanged after the complexation to the metal ion and thus does not have an influence on the coordination to the metal center [51].

### Electronic spectra, magnetic moments, and molar conductivity measurement

**Fig. 1:**
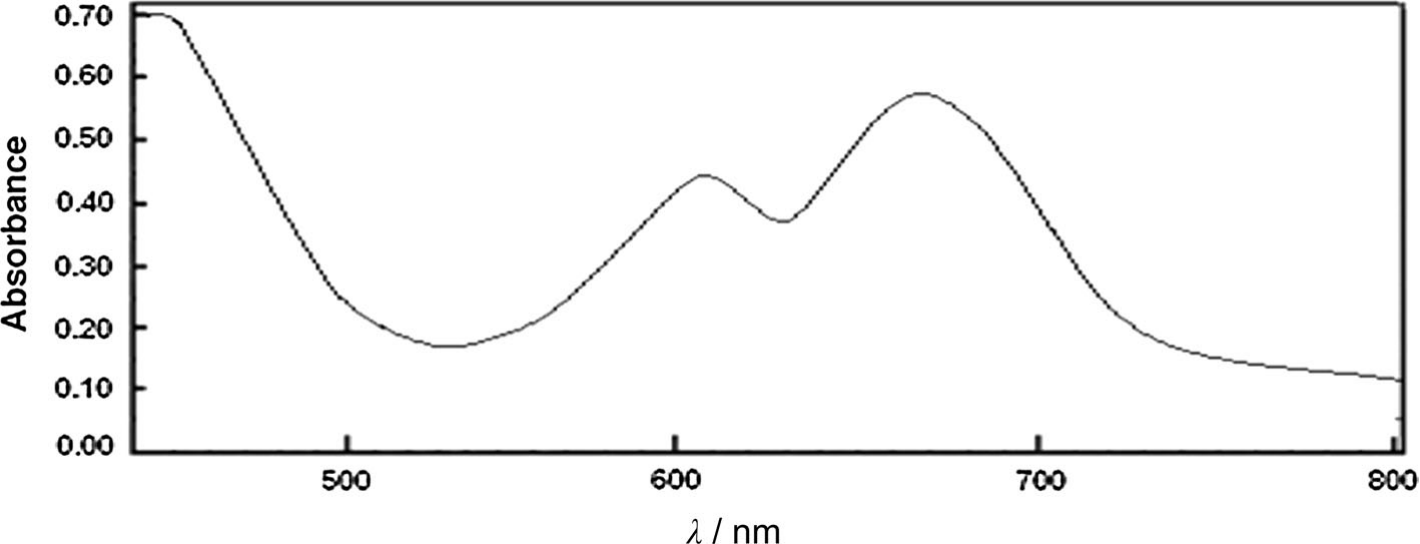
Electronic absorption spectrum of **1** in a DMF solution (10^−3^ M)

The electronic spectra of the ligand and its transition metal complexes in a DMF solution (10^−3^ M) with their assignments, magnetic moments, and molar conductivity measurements are given in Table 1. The electronic spectrum of H_4_L shows absorption bands at 270 and 316 nm, which are assigned to π–π* transitions within the coumarin moiety [52]. The broad band at 365 nm is attributed to the π–π* transitions of the C=N and C=O bonds in addition to a broad band at 410 nm, attributed to the *n*–π* transition, which is overlapping with the intramolecular CT-band of the phenyl ring to the azomethine group. **1** shows two well-resolved peaks at 675 nm, attributed to the ^4^T_1_(P) ← ^4^A_2_(F) transition and at 610 nm, assigned to the ^4^A_2_ → ^4^T_1_(F) transition. These transitions, together with the magnetic moment of the complex (*μ* = 4.22 B.M.), reveal that the geometry around the Co(II) ion is tetrahedral. Figure 1 shows the electronic absorption spectrum of **1**. The exact value of its magnetic moment supports the previous conclusion. The molecular ion peak was observed at *m/z* = 618, confirming the molecular weight. The mass spectrum and the schematic fragmentation can be seen in the supplementary materials S4.

The electronic spectrum of **6** shows two bands, corresponding to the ^4^A_2g_(F) ← ^4^T_1g_(F) and the ^4^T_1g_(P) ← ^4^T_1g_(F) transition at 575 and 660 nm, respectively [53]. These transitions are consistent with the formation of an octahedral high-spin geometry. The measured magnetic moment of this complex indicates an octahedral geometry. The exact value of its magnetic moment is 4.69 B.M., supporting the previous conclusion. The mass spectrum of the complex shows its molecular ion peak at *m/z* = 737, which is consistent with the proposed molecular weight. The mass spectrum of **6** and the schematic fragmentation can be seen in the supplementary materials S5.

**2** shows a magnetic moment of 2.92 B.M., corresponding to two unpaired electrons. The electronic spectrum displays bands at 652 and 766 nm. These bands may be assigned to the ^3^A_2_(F) → ^3^T_2_(F)(*ν*_1_) and the ^3^A_2_(F) → ^3^T_1_(F)(*ν*_2_) transitions, respectively. It suggests a tetrahedral geometry of the Ni(II) complex [54]. The magnetic moment of this complex was measured and equals 3.32 B.M., which lies in the typical range for a tetrahedral complex. The electronic spectrum of **7** indicates an octahedral geometry of the complex. The main bands at 652 and 435 nm are attributed to the ^3^T_1g_(F) ← ^3^A_2g_(F) and the ^3^T_1g_ ← ^3^A_2g_(F) transitions, respectively. Another band, attributed to the ^3^T_1g_ ← ^3^A_2g_(F) transition, was expected, but not observed, as it lies in the near infrared range. The magnetic moment was measured and found to be 3.29 B.M., which confirms the previous geometry.

**Fig. 2:**
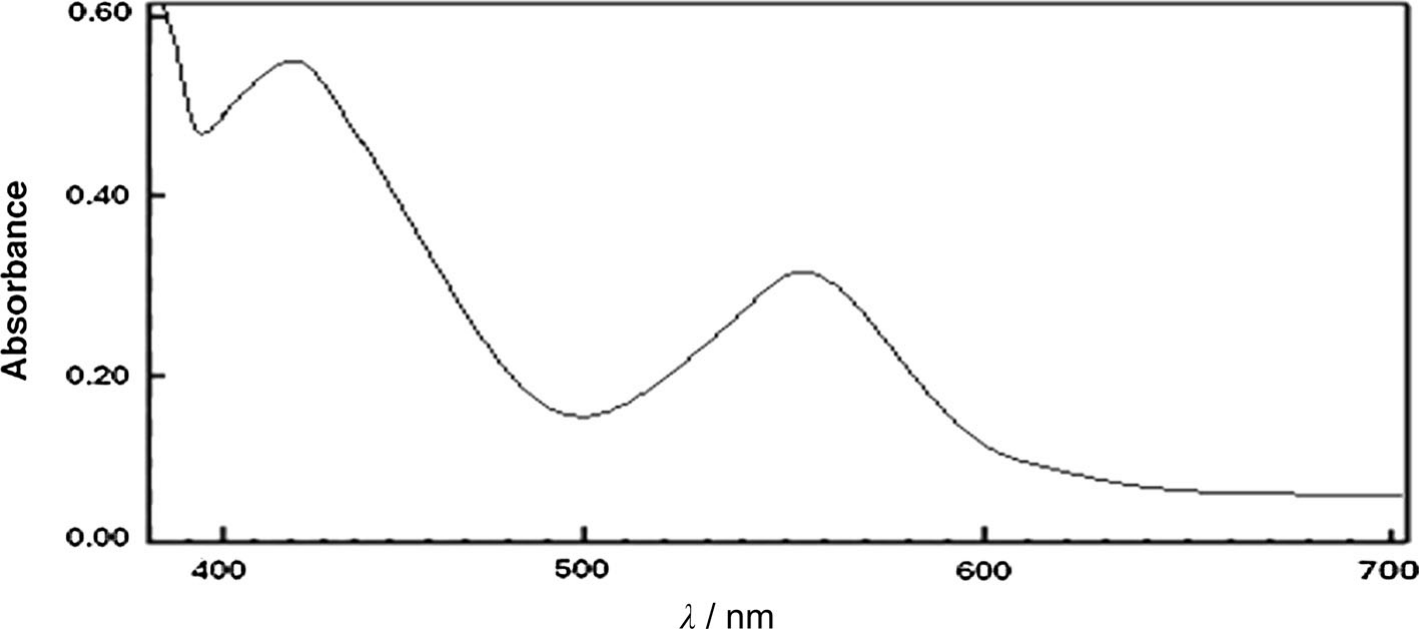
Electronic absorption spectrum of **3** in a DMF solution (10^−3^ M)

The electronic spectrum of **3** shows two bands at 439 and 567 nm, corresponding to the ^2^B_1g_ → ^2^E_g_ and the ^2^B_1g_ → ^2^A_1g_ transitions in a square planar geometry (Fig. 2). The value of the magnetic moment is 1.97 B.M., which lies within the possible range, recorded for complexes with one unpaired electron [55]. A square pyramidal geometry is proposed for the complex **8** based on the presence of two bands at 455 and 610 nm. These bands may be assigned to the ^2^B_1_ ← ^2^A_1_ and the ^2^B_1_ ← ^2^E transitions, respectively, based on assignments published before [56, 57]. The value of the magnetic moment is in accordance with the previous results (1.83 BM).

The reflectance spectra of the oxovanadium complexes **4** and **9** reveal three bands at 362, 613, and 754 nm for **7** and 436, 624, and 775 nm for **9**, which is assigned to the ^2^B_2_ → ^2^E, the ^2^B_2_ → ^2^B_1_, and the ^2^B_2_ → ^2^A_1_ transitions for a distorted square-pyramid geometry. The magnetic moment was found to be 1.81 and 1.74 B.M. for the complexes **7** and **9**, respectively [58].

The electronic spectra of the complexes **5** and **10** show two bands in the range of 453–520 and 720–765 nm, respectively. The first band is assigned to charge transfer transitions from the out-of-plane pπ orbital of the phenolate coumarin oxygen to the half-filled *d*_*x*2–*y*2_/*d*_*z*2_ orbital of the high-spin Fe(III) [59]. Elemental analysis and the infrared spectra give significant information about the coordination mode of the nitrate anion as a bi- and monodentate anion for the complexes **5** and **10**, respectively. On the other hand, the magnetic moments of the Fe(III) complexes **5** and **10** were measured and their values are 4.35 and 4.87 B.M., respectively, in accordance with a high-spin distorted octahedral arrangement (t_2g_^3^e_g2_).

The presented magnetic moments do not show any metal–metal interactions in the bimetallic complexes. Very weak antiferromagnetic interactions cannot be excluded, for this low temperature (He-temperature) measurements of the magnetic moment would be needed, which are out of our reach. Otherwise, this would be speculation.

Molar conductivities of the complexes were recorded in DMF (1.0 × 10^−3^ M). It has been reported that DMF is a good donor solvent, since it can replace NO_3_^−^ in metal complexes [60]. Conductivity measurements indicate the non-electrolytic nature of the complexes **3**, **4**, and **9**. Because the bi-coordinated binding of SO_4_^2−^ is usually stronger, DMF will not replace SO_4_^2−^. In other words, the Lewis basicity (donor number) of DMF is not big enough to replace SO_4_^2−^ in complex **9** [61]. Complexes **1** and **2** exhibit molar conductivities of 95 and 92 Ohm^−1^ cm^2^ mol^−1^, respectively, and can therefore be considered as 1:1 electrolytes, since a maximum value about 100 Ohm^−1^ cm^2^ mol^−1^ is reported for 1:1 electrolytes in DMF [60]. On the other hand, the complexes **5**, **6**, **7**, **8**, and **10** behave as 1:2 electrolytes.

### ESR spectroscopy

**Fig. 3:**
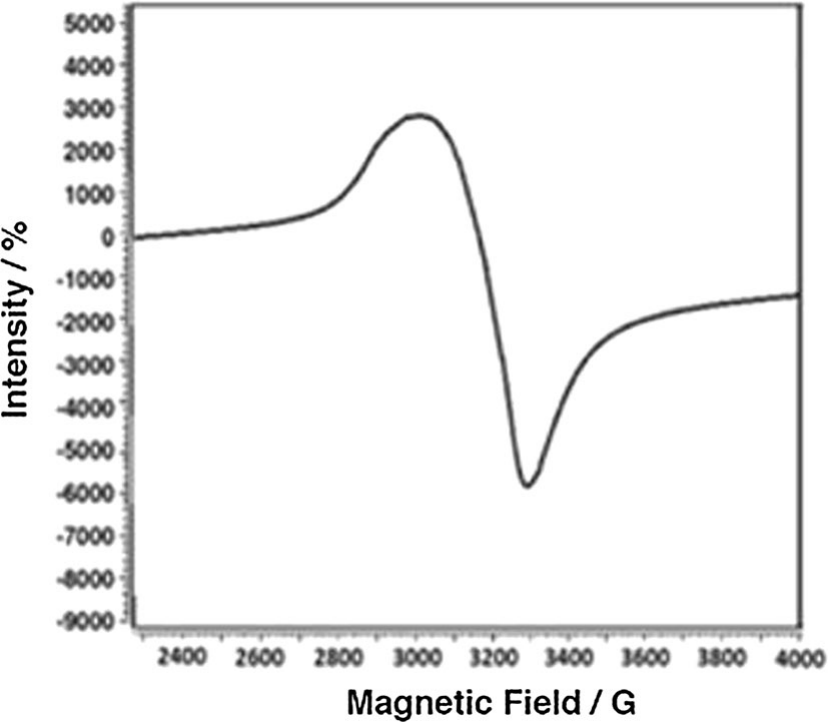
ESR spectrum of **3**

**Fig. 4:**
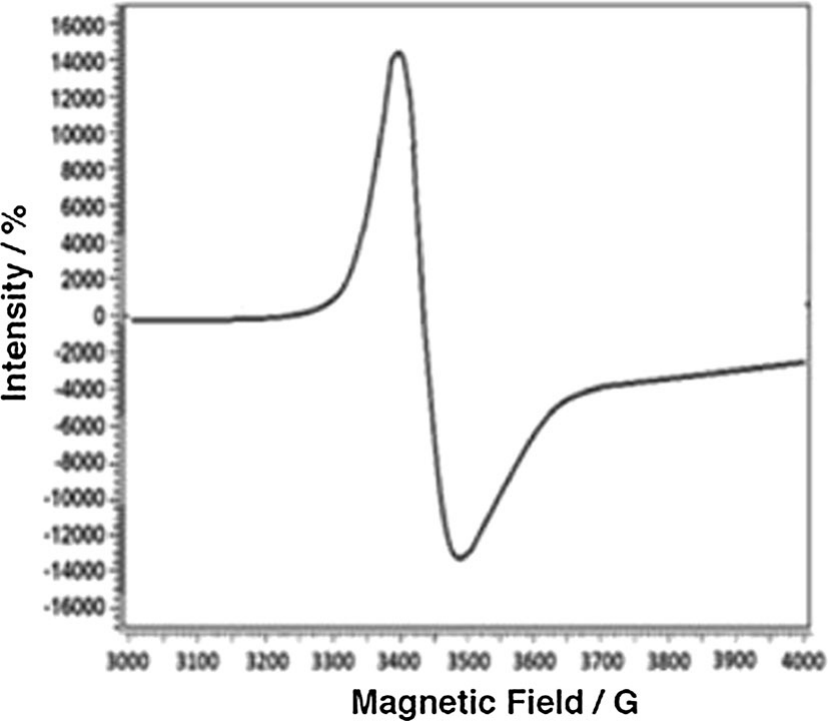
ESR spectrum of **9**

X-band ESR spectra were recorded in the solid state for the complexes **3** (Fig. 3) and **9** (Fig. 4) at 25 °C. The g-tensor values of the Cu(II) complex can be used to derive the ground state. The observed values, *g*∥(2.136) > *g*⊥ (2.053) > *g*_e_(2.0023), indicate that the copper site has a *d*_*x*2−*y*2_ orbital giving ^2^B_1g_ as the ground state, which is characteristic for a square planar or an octahedral geometry [62]. The *g*║ value is an important value, when it comes to indicating the covalent or ionic character of the M–L bond. Kivelson and Neiman reported for an ionic character of the bond *g*║ > 2.3 and for a covalent character *g*║ < 2.3. In the present complex, *g*║ is smaller than 2.3, which indicates a covalent character of the Cu–L bond [63]. The axial symmetry parameter *G* is smaller than four, which indicates a considerable exchange interaction in the solid complex [14]. If the value of *G* is less than 4, a considerable exchange interaction is noticed in the solid complex. The calculated *G* value of the square planar complex **3** suggests a strong interaction between the Cu(II) centers [64]. The X-band ESR spectrum of **9** gives a broad peak without hyperfine coupling, where the *g*-tenser values are *g*∥(1.99) and *g*⊥ (1.97). The decrease of the *g* values compared to the value of a free-electron (2.0023) could be attributed to the ligand field strength. The shape of the spectrum, as well as the *g*-tenser values agrees with a square-pyramidal geometry for the vanadyl complex **9**.

### Thermal analysis

Thermal gravimetric analysis was carried out for selected complexes **4**, **5**, and **10**, to gain information concerning their thermal stability and to decide, whether water molecules are bound in the inner or outer coordination sphere of the central metal ions [65]. The TGA curves showed that the decomposition of the complexes proceeds in many steps in the weight loss percentage. The first stage is the weight loss, which corresponds to the loss of lattice or coordinated water molecules. The second stage corresponds to the loss of nitrate ions in the form of N_2_O_5_ gas, followed by the organic ligand. At the end, metal oxide was formed around the range from 700 to 1000 °C, where residues are nearly close to the calculated values. In Table 2, the thermal analysis is summarized. The degradation and the schematic fragmentation pathways of the complexes are shown in detail in the supplementary material S6.

**Table 2:** Results of the thermal analysis of complexes **4**, **5**, and **10**

Complex	Decomposition transitions	*M*/g mol^−1^	Temperature/°C		Weight loss/%	
			TGA	DrTGA	Found	Calculated
**4**	(I) [(VO)_2_(H_2_L)_2_]·2H_2_O → (− 2H_2_O)	1090.63	60–136	95	4.10	3.32
	(II) [(VO)_2_(H_2_L)_2_] → (− 2(H_2_L))		136–668	326	80.30	79.93
	(III) Complete decomposition		668–1000	630	17.81	16.76
**5**	(I) Fe_2_(H_2_L)_2_(NO_3_)_2_(H_2_O)_2_ → (−2 H_2_O)	1192.44	142–254	123	3.58	3.02
	(II) Fe_2_(H_2_L)_2_(NO_3_)_2_ → (− N_2_O_5_)		254–381	310	9.32	9.05
	(III) Fe_2_(H_2_L)_2_O → (− 2(H_2_L))		381–760	623	76.31	74.54
**10**	(I) Fe_2_(L)(H_2_O)_4_(NO_3_)_2_ → (−4 H_2_O)	766.10	161–266	134	10.13	9.31
	(II) Fe_2_(L)(NO_3_)_2_ → (− N_2_O_5_)		266–393	216	15.82	14.10
	(III) Fe_2_(L)O → (− L)		393–745	526	51.60	55.61

### Cyclic voltammetry studies

**Fig. 5:**
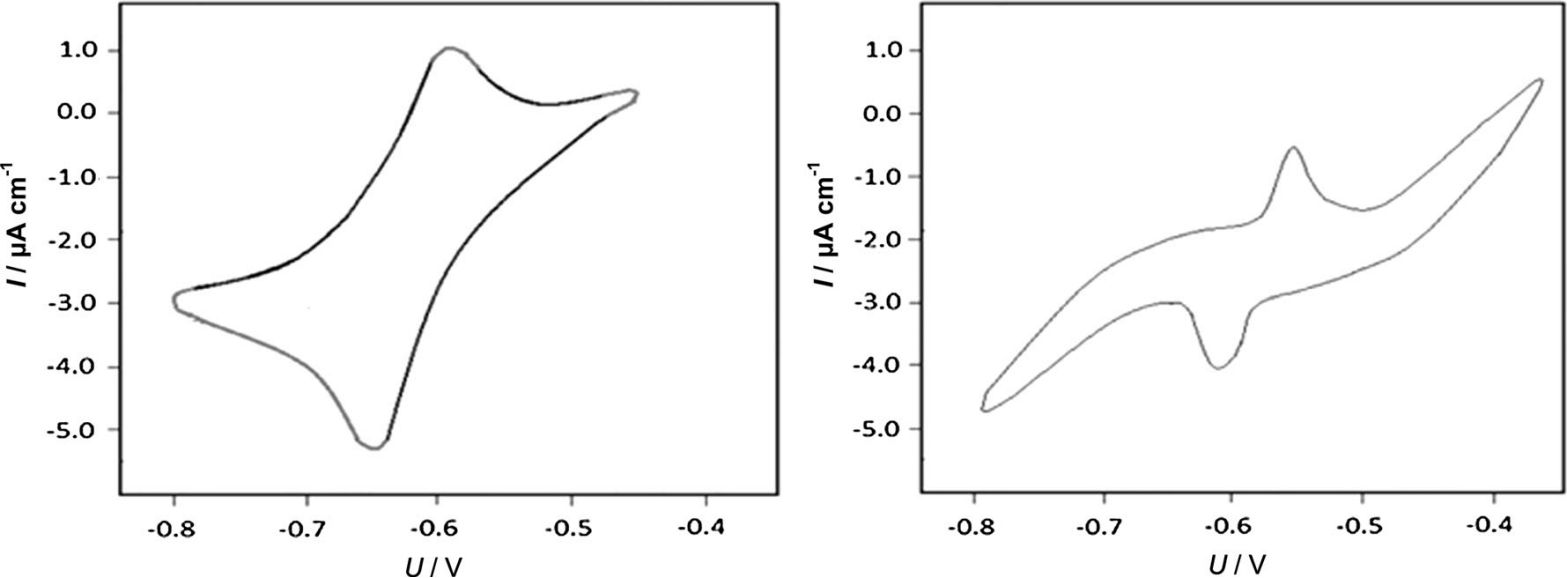
Cyclic voltammograms of **1** (left), **2** (right)

The electrochemical properties of the metal complexes have been studied, to determine the structural changes accompanying with the electron transfer. The redox behavior has been investigated for **1** and **2** in DMSO (1.0 mM dm^−3^). The measurements were carried out in a 0.1 M tetrabutylammonium tetraflouroborate (TBA + BF_4_^−^) solution as a supporting electrolyte, using platinum wires with a diameter of 0.5 mm as working and counter electrodes, and Ag/AgCl as a reference electrode. Ferrocene/ferrocenium (Fc/Fc^+^) was used as internal standard for the assignment of the potential electrode couple [41, 42]. The complexes **1** and **2** show electrochemical reversible steps (respective pseudo-reversible), related to single-electron transfer processes [66, 67]. The cyclic voltammogram of the Co(II) complex (Fig. 5, left) refers to *E*_1/2_ = − 0.640 V. The Ni(II) complex (Fig. 5, right) refers to *E*_1/2_ = − 0.591 V, both corresponding to the M(II)/M(III) one-electron redox system. The ratio *I*_pa_/*I*_pc_ is close to unity.

From the interpretation of elemental analysis, magnetic studies and spectral data, as well as the thermal analysis and molar conductivity measurements, one can propose tentative structures of the synthesized transition metal complexes. Scheme 1 depicts the suggested structures for the obtained metal complexes.

**Figure:**
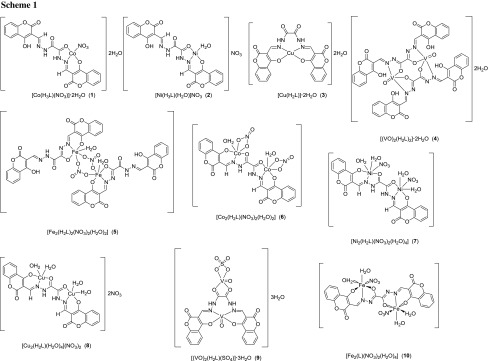

### Biological studies

**Fig. 6:**
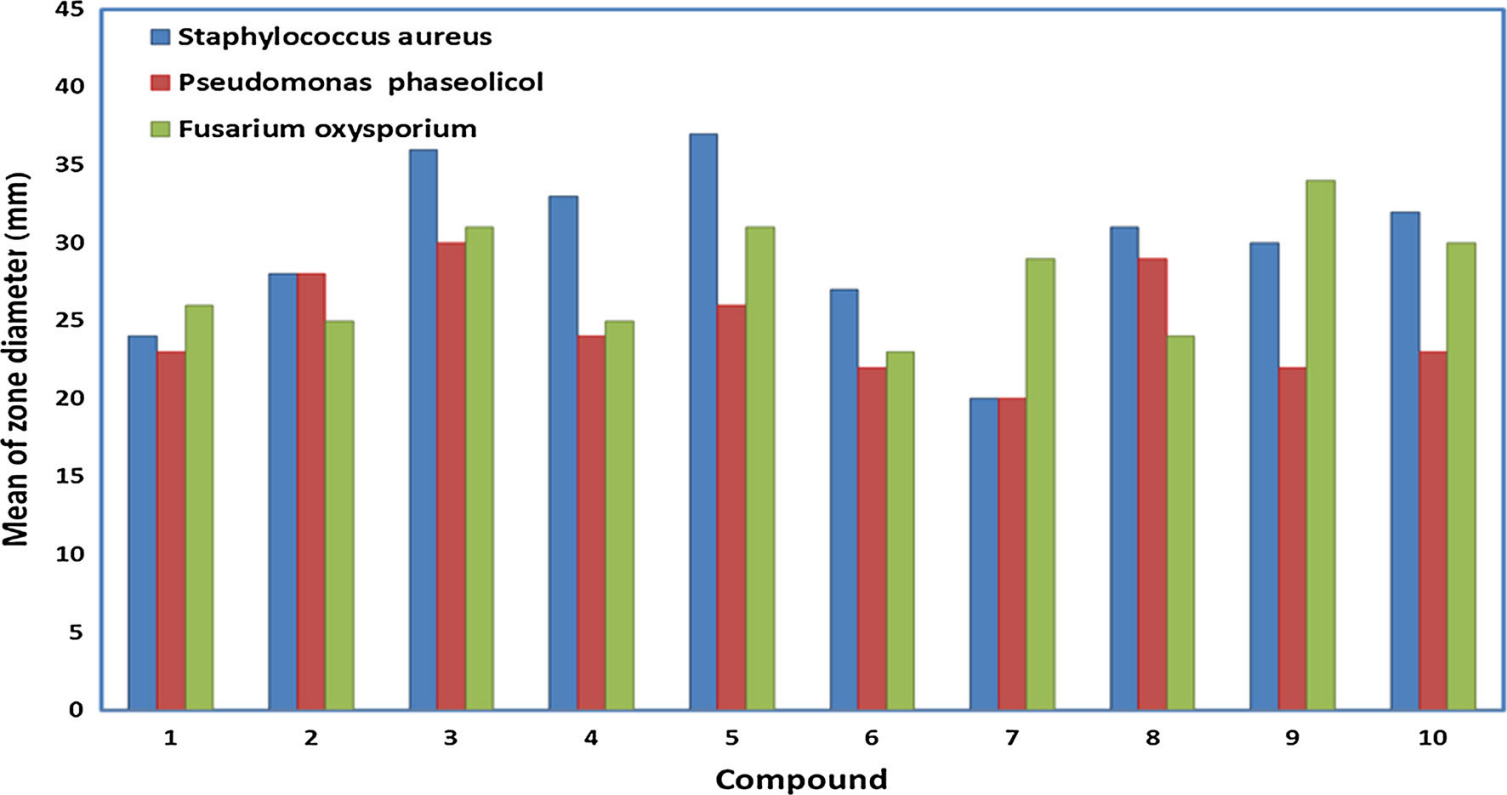
Biological screening of the ligand and its complexes against Gram-positive bacteria, Gram-negative bacteria, and fungi

The Schiff base ligand H_4_L and its metal complexes were examined according to antimicrobial activity against Gram-positive bacteria (*Staphylococcus aureus*) and Gram-negative bacteria (*Pseudomonas fluorescens*), as well as one pathogenic fungus (*Fusarium oxysporum*). The results of the biological studies of the ligand and its complexes are given in Fig. 6 and Table 3. The data is compared to standard antibiotics, chloramphenicol, as standard reference for Gram-negative and cephalothin for Gram-positive bacteria. Cycloheximide was used as antifungal standard reference. The in vitro antibacterial and antifungal activities demonstrate that the complexes have higher antimicrobial activities compared to that of the ligand. This behavior could explain that the chelation tends to make the ligand act as a more powerful and potent antibacterial agent. Tweedy’s theory [68] suggests that the chelation reduces the polarity of the metal atom due to the partial sharing of its positive charge with a donor group and thus the possible π-electron delocalization over the whole chelate ring is enabled [69]. Such a chelation could enhance the lipophilic character of the central metal atom. Subsequently, this favors its permeation through the lipid layers of the cell membrane and thus, bears the possibility to block the metal binding site in the enzyme of a microorganism [70]. The variety in the activity of different complexes against various microorganisms depends either on the cells, the impermeability of the microbes, or the contrasts of ribosomes in microbial cells. There are other factors, which increase the activity, like solubility, conductivity, and bond length between the metal and the ligand. The results also reveal that Cu(II), Fe(III), and VO^2+^ complexes display the highest (significant) inhibition against the growth of the selected bacteria and fungi. On the other hand, Co(II) and Ni(II) complexes show moderate activity. According to the data, there is an evidence for the relationship of the structure of the complexes and their activity, where the antimicrobial activity is enhanced by binuclear complexes, rather than by acyclic complexes, which reveals that these complexes are biologically more efficient and thus, provide the possibility to be useful as new drugs and discuss that the chemical geometry of compounds is important to explain the biological activity of the complexes.

**Table 3:** Antimicrobial activity of the Schiff base ligand H_4_L and its metal complexes

Compound	Mean of zone diameter/mm mg cm^−3^		
	Gram—positive bacteria	Gram—negative bacteria	Fungi
	*Staphylococcus aureus*	*Pseudomonas phaseolicol*	*Fusarium oxysporium*
H_4_L	$22 \pm \, 0.2$	13 $\pm \, 0.1$	17 $\pm \, 0.2$
**1** [Co(H_3_L)(NO_3_)]·2H_2_O	24 $\pm \, {\kern 1pt} 0.6$	23 $\pm \, 0.1$	26 $\pm \, 0.2$
**2** [Ni(H_3_L)(H_2_O)]NO_3_	28 $\pm \, {\kern 1pt} 0.2$	28 $\pm \, 0.3$	25 $\pm \, 0.2$
**3** [Cu(H_2_L)]·2H_2_O	36 $\pm \, {\kern 1pt} 0.3$	30 $\pm \, 0.2$	31 $\pm \, 0.3$
**4** [(VO)_2_(H_2_L)_2_]·2H_2_O	33 $\pm \, {\kern 1pt} 0.1$	24 $\pm \, 0.3$	25 $\pm \, 0.3$
**5** Fe_2_(H_2_L)_2_(NO_3_)_2_(H_2_O)_2_	37 $\pm \, 0.4$	26 $\pm \, 0.1$	31 $\pm \, 0.2$
**6** Co_2_(H_2_L)(NO_3_)_2_(H_2_O)_2_	27 $\pm \, 0.4$	22 $\pm \, 0.1$	23 ± 0.2
**7** Ni_2_(H_2_L)(NO_3_)_2_(H_2_O)_4_	20 $\pm \, 0.6$	20 $\pm \, 0.1$	29 $\pm \, 0.3$
**8** [Cu_2_(H_2_L)(H_2_O)_4_](NO_3_)_2_	31 $\pm \, 0.1$	29 $\pm \, 0.2$	24 $\pm \, 0.3$
**9** [(VO)_2_(H_2_L)(SO_4_)]·3H_2_O	30 $\pm \, 0.1$	22 ± 0.2	34 $\pm \, 0.3$
**10** Fe_2_(L)(H_2_O)_4_(NO_3_)_2_	32 $\pm \, 0.2$	23 $\pm \, 0.1$	30 $\pm \, 0.2$
Antibiotic	42	36	40

## Conclusion

The hydrazone Schiff base ligand acts as hexa-, penta-, tetra- or monodentate ligand with metal cations to afford a series of mono or binuclear complexes. It is known from IR spectra that the chelation of all acyclic divalent metal ions to the ligand occurs via the phenolic atoms of the coumarin moiety, the ketonic oxygens, and the nitrogen atoms of the azomethine groups, except for **4**, **5**, and **10**, where the ligand coordinates in its enol form. Nitrate, sulfate, and chloride ions in addition to water molecules, which can be crystalline or coordinated, satisfy the metal’s coordination sites and thus, bear the possibility to complete the metal’s geometry around its center. The spectroscopic studies and the molar conductivity measurements of all the complexes were used to examine the type of the coordination and the metal’s geometry around its center. Mono- and binuclear complexes exhibit either tetrahedral, square planar, square pyramidal, or octahedral geometries. The synthesized Schiff base and the corresponding metal complexes were tested for the growth inhibitory activity against phytopathogenic bacteria and fungi. It is obvious that the metal complexes are more toxic against bacteria and fungi in comparison with their ligand. Cyclic voltammograms of the complexes **1** and **2** show one-electron transfer processes, indicating their electro-activity in solution.

### Experimental

The nitrate salts of cobalt(II), nickel(II), iron(III), and copper(II) were purchased from Merck or DBH, vanadyl(IV) sulfate pentahydrate from Sigma Aldrich. Organic solvents [absolute ethyl alcohol, methyl alcohol, acetone, dimethylformamide (DMF), and dimethylsulfoxide (DMSO)] were reagent grade and used without further purification.

Microanalyses of carbon, hydrogen, and nitrogen were carried out on a Perkin-Elmer 2400 Series II Analyzer. Electronic spectra of the metal complexes in DMF were carried out on a UV–Vis Perkin-Elmer Model Lamda 900. NIR-, IR- and mid-range FT-IR spectra of the compounds were recorded as KBr pellets within the range of 400–4000 cm^−1^, using a Perkin–Elmer 16PC FT-IR spectrometer. Far FT-IR spectra were recorded within the range of 600–200 cm^−1^ on a Perkin–Elmer System 2000 spectrometer as polyethylene pellets. Analyses of the metals in the complexes were carried out according to the standard method [71]. Mass spectra were recorded on a Shimadzu-GC–MS–QP mass spectrometer (model 1000 EX) using a direct inlet system at 220 °C and 70 eV. NMR spectra of the ligand were carried out in DMSO-*d*_6_ on a Bruker WP 200 SY spectrometer at room temperature using TMS as an internal standard. Magnetic susceptibilities of the complexes were measured at room temperature using a Johnson Matthey, Alfa Products, model MKI magnetic susceptibility balance. The effective magnetic moments were calculated from the expression *μ*_eff_ = 2.828 (*χ*_M.T_)1/2 B.M., where *χ*_M_ is the molar susceptibility, corrected using the Pascal’s constants for the diamagnetism of all atoms in the compounds [72]. Melting points were measured using a Stuart melting point instrument. ESR spectra of the copper complexes were recorded on a Bruker BioSpin spectrometer. The molar conductance of 1.0 × 10^−3^ M solution in DMF was measured on a WTW.D8120 Weilheim L.F.42 conductivity meter. TGA curves were obtained using a NETZSCH-Gerätebau. Thermal gravimetric analysis (TGA) was carried out in a range from room temperature up to 1000 °C with a heating rate of 10 °C min^−1^ in a nitrogen atmosphere. The cyclic voltammetry measurements were carried out with a Potentiostat Wave Generator (Oxford press), and equipped with a Phillips PM 8043 X–Y recorder. The electrochemical cell assembly consists of platinum wires of 0.5 mm diameter as working and counter electrodes, and Ag/AgCl as a reference electrode.

#### Ethanedioic acid 1,2-bis[(4-hydroxy-3-methylene-2H-1-benzopyran)hydrazide] (H_4_L, C_22_H_14_N_4_O_8_)

In a first step, 4-hydroxycoumarin was reacted with DMF to obtain 3-formyl-4-hydroxycoumarin [73]. The final Schiff base ligand H_4_L was then prepared by the addition of a solution of oxalic dihydrazide (2.54 mmol) in 40 cm^3^ ethanol/H_2_O (70%/30%, v/v) to 3-formyl-4-hydroxycoumarin (5.05 mmol) in 40 cm^3^ ethanol. The reaction mixture was refluxed for 3 h. After the solution was slowly cooled to room temperature, yellow crystals were formed, which were filtered off and washed with ethanol and diethyl ether, yielding 0.89 g (70.63%) of the title substance (Scheme 2). M.p.: 174 °C; ^1^H NMR (200 MHz, DMSO-*d*_6_): *δ* = 12.43 (s, 2H, e, e′), 10.21 (s, 2H, g, g′), 8.32 (s, 2H, f, f′), 7.64 (t, 2H, c, c′), 7.43 (d, 2H, a, a′), 7.34 (d, 2H, d, d′), 7.31 (t, 2H, b, b′) ppm; ^13^C NMR (50 MHz, DMSO-*d*_6_): *δ* = 165.69 (s), 161.97 (s), 153.56 (s), 151.12 (s), 149.03 (s), 132.71 (s), 123.94 (s), 123.24 (s), 116.40 (s), 115.84 (s), 91.05 (s) ppm; IR (KBr): $\bar{\nu }$ = 3376 (OH), 3142–3180 (NH), 1695 (C=O), 1635 (C=N) cm^−1^; UV–Vis (DMF, *c* = 10^−3^ mol dm^−3^): *λ*_max_ = 470, 410, 365, 316 nm; MS (70 eV): *m*/*z* = 371 (M^+^).

**Figure:**
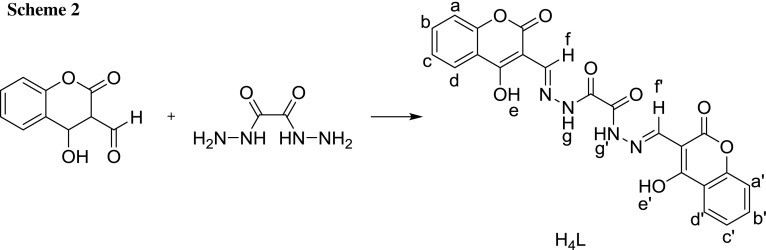

### Synthesis of the transition metal complexes

The coordination reactions of the Schiff base ligand H_4_L to cobalt(II), nickel(II), copper(II), oxovanadium(IV), and iron(III) were carried out in the molar ratios of 1:1 (ligand:metal), whereby mono- and binuclear transition metal complexes were formed. The ligand itself could act in its mono-, di-, tri- or even tetra-deprotonated form, whereby lithium hydroxide monohydrate was added in the molar ratio of 2:1 (LiOH·H_2_O:H_4_L), to facilitate the ligand’s deprotonation. However, trials to prepare mononuclear complexes of VO(IV) and Fe(III) of H_4_L were not successful. Both metal ions formed binuclear complexes. This behavior could be proved also by elemental analysis. The existence of numerous coordination sites of H_4_L bears the possibility of variable binding modes. Thus, a great number of mono- and binuclear transition metal complexes could be obtained. The complexes are air stable in the solid state and soluble in DMF and DMSO.

#### (*O*,*N*,*O*)(Ethanedion-acido,1,2-bis[(4-hydroxy-3-methylene-2H-1-benzopyran)hydrazide])cobalt(II)nitrate dihydrate ([Co(H_3_L)(NO_3_)]·2H_2_O, **1**, C_22_H_17_CoN_5_O_13_)

A solution of Co(NO_3_)_2_·6H_2_O (8.59 mmol) in 20 cm^3^ ethanol was added gradually to a solution of the prior with LiOH·H_2_O deprotonated ligand (L^4−^) (8.59 mmol) in 40 cm^3^ ethanol. The reaction mixture was stirred for 30 min and then refluxed for 2 h. The precipitate of the desired complex was formed already at reflux temperature. The formed complex was filtered off, washed with ethanol and diethyl ether, and finally air-dried, yielding 2.97 g (56%) of a brownish blue powder. D.p.: > 250 °C; IR (KBr): $\bar{\nu }$ = 3358 (OH), 3162 (NH), 1696 and 1685 (C=O), 1635 and 1624 (C=N), 372 (Co–O), 444 (Co–N) cm^−1^; UV–Vis (DMF, *c* = 10^−3^ mol dm^−3^): *λ*_max_ = 610, 675 nm; MS (70 eV): *m*/*z* = 618 (M^+^).

#### (*O,N,O*)(Ethanedion-acido,1,2-bis[(4-hydroxy-3-methylene-2H-1-benzopyran)hydrazide])(aqua)nickel(II)nitrate ([Ni(HL)(H_2_O)]NO_3_, **2**, C_22_H_15_N_5_NiO_12_)

**2** was prepared in the same way as **1**, using Ni(NO_3_)_2_·6H_2_O (8.59 mmol) as precursor. Yield: 2.63 g (51%) of a brownish red powder. D.p.: > 250 °C; IR (KBr): $\bar{\nu }$ = 3374 (OH), 3156 (NH), 1639 and 1681 (C=O), 1632 and 1618 (C=N), 572 (Ni–O), 426 (Ni–N) cm^−1^; UV–Vis (DMF, *c* = 10^−3^ mol dm^−3^): *λ*_max_ = 652, 766 nm.

#### (*O,N,N,O*)(Ethanedion-acido,1,2-bis[(4-hydroxy-3-methylene-2H-1-benzopyran)hydrazide])copper(II) dihydrate ([Cu(H_2_L)]·2H_2_O, **3**, C_22_H_16_CuN_4_O_10_)

**3** was prepared in the same way as **1**, using Cu(NO_3_)_2_·6H_2_O (8.59 mmol) as precursor. Yield: 3.41 g (71%) of a green powder. D.p.: > 250 °C; IR (KBr): $\bar{\nu }$ = 3368 (OH), 3160 (NH), 1694 (C=O), 1624 (C=N), 584 (Cu–O), 458 (Cu–N) cm^−1^; UV–Vis (DMF, *c* = 10^−3^ mol dm^−3^): *λ*_max_ = 439, 567 nm.

#### Bis(*N,N′,O,O′*)(Ethanedion-acido,1,2-bis[(4-hydroxy-3-methylene-2H-1-benzopyran)hydrazide])(oxo)vanadium(IV) dihydrate ([(VO)_2_(H_2_L)_2_]·2H_2_O, **4**, C_44_H_28_N_8_O_20_V_2_)

**4** was prepared in the same way as **1**, using VOSO_4_·5H_2_O (8.59 mmol) as precursor. Yield: 5.06 g (54%) of a green powder. D.p.: > 250 °C; IR (KBr): $\bar{\nu }$ = 3355 (OH), 1632 and 1616 (C=N), 562 (V–O), 488 (V–N) cm^−1^; UV–Vis (DMF, *c* = 10^−3^ mol dm^−3^): *λ*_max_ = 613, 754 nm.

#### Bis(*O,N,O*)(Ethanedion-acido,1,2-bis[(4-hydroxy-3-methylene-2H-1-benzopyran)hydrazide])(μ-*O,O*-dinitrato)(aqua)iron(III) (Fe_2_(H_2_L)_2_(NO_3_)_2_(H_2_O)_2_, **5**, C_44_H_28_Fe_2_N_10_O_24_)

A solution of the prior with LiOH·H_2_O deprotonated ligand (L^4−^) (2.152 mmol) in 20 cm^3^ ethanol was added slowly to a solution of Fe(NO_3_)_3_·6H_2_O (2.152 mmol) in 40 cm^3^ ethanol. The reaction mixture was first stirred for 30 min and refluxed for 6 h. The precipitate of the desired metal complex was formed, filtered off, washed with ethanol and diethyl ether, and finally air-dried. Yield: 1.80 g (70%) of a black powder. D.p.: > 250 °C; IR (KBr): $\bar{\nu }$ = 3417 (OH), 3160 (NH), 1682 (C=O), 1632 and 1623 (C=N), 542 (Fe–O), 456 (Fe–N) cm^−1^; UV–Vis (DMF, *c* = 10^−3^ mol dm^−3^): *λ*_max_ = 553, 720 nm.

#### (*O,N,O*)(Ethanedion-acido,1,2-bis[(4-hydroxy-3-methylene-2H-1-benzopyran)hydrazide])(κ-*O,O*-dinitrato)(diaqua)dicobalt(II) (Co_2_(H_2_L)(NO_3_)_2_(H_2_O)_2_, **6**, C_22_H_16_Co_2_N_6_O_16_)

**6** was prepared in the same way as **5**, using Co(NO_3_)_2_·6H_2_O (2.15 mmol) as precursor. Yield: 0.97 g (61%) of a brown powder. D.p.: > 250 °C; IR (KBr): $\bar{\nu }$ = 3374 (OH), 3152 (NH), 1680 (C=O), 1615 (C=N), 544 (Co–O), 466 (Co–N) cm^−1^; UV–Vis (DMF, *c* = 10^−3^ mol dm^−3^): *λ*_max_ = 575, 660 nm; MS (70 eV): *m*/*z* = 737 (M^+^).

#### (*O,N,O*)(Ethanedion-acido,1,2-bis[(4-hydroxy-3-methylene-2H-1-benzopyran)hydrazide])(dinitrato)(tetraaqua)dinickel(II) (Ni_2_(H_2_L)(NO_3_)_2_(H_2_O)_4_, **7**, C_22_H_20_N_6_Ni_2_O_18_)

**7** was prepared in the same way as **5**, using Ni(NO_3_)_2_·6H_2_O (2.15 mmol) as precursor. Yield: 0.93 g (56%) of a green powder. D.p.: > 250 °C; IR (KBr): $\bar{\nu }$ = 3368 (OH), 3151 (NH), 1678 (C=O), 1621 (C=N), 562 (Ni–O), 458 (Ni–N) cm^−1^; UV–Vis (DMF, *c* = 10^−3^ mol dm^−3^): *λ*_max_ = 652, 435 nm.

#### (*O,N,O*)(Ethanedion-acido,1,2-bis[(4-hydroxy-3-methylene-2H-1-benzopyran)hydrazide])(tetraaqua)dicopper(II)nitrate ([Cu_2_(H_2_L)(H_2_O)_4_](NO_3_)_2_, **8**, C_22_H_20_Cu_2_N_6_O_18_)

**8** was prepared in the same way as **5**, using Cu(NO_3_)_2_·6H_2_O (2.15 mmol) as precursor. Yield: 1.05 g (62%) of a violet powder. D.p.: > 250 °C; IR (KBr): $\bar{\nu }$ = 3481 (OH), 3170 (NH), 1663 (C=O), 1624 (C=N), 567 (Cu–O), 473 (Cu–N) cm^−1^; UV–Vis (DMF, *c* = 10^−3^ mol dm^−3^): *λ*_max_ = 610, 455 nm.

#### *(N,N,O,O,O,O)*(Ethanedion-acido,1,2-bis[(4-hydroxy-3-methylene-2H-1-benzopyran)hydrazide])(κ-*O,O*-sulfato)(dioxo)divanadium(IV) trihydrate ([(VO)_2_(H_2_L)(SO_4_)]·3H_2_O, **9**, C_22_H_18_N_4_O_17_SV_2_)

**9** was prepared in the same way as **5**, using VOSO_4_·5H_2_O (2.15 mmol) as precursor. Yield: 1.14 g (71%) of a green powder. D.p.: > 250 °C; IR (KBr): $\bar{\nu }$ = 3433 (OH), 3167 (NH), 1675 (C=O), 1615 (C=N), 582 (V–O), 486 (V–N) cm^−1^; UV–Vis (DMF, *c* = 10^−3^ mol dm^−3^): *λ*_max_ = 624, 775 nm.

#### (*O,N,O*)(Ethanedion-acido,1,2-bis[(4-hydroxy-3-methylene-2H-1-benzopyran)hydrazide])(dinitrato)(tetraaqua)diiron(III) (Fe_2_(L)(H_2_O)_4_(NO_3_)_2_, **10**, C_22_H_18_Fe_2_N_6_O_18_)

A solution of H_4_L (1.076 mmol) in 20 cm^3^ ethanol was added slowly to a solution of Fe(NO_3_)_3_·6H_2_O (2.152 mmol) in 40 cm^3^ ethanol. The reaction mixture was first stirred for 30 min and refluxed for 6 h. The precipitate of the desired metal complex was formed, filtered off, washed with ethanol and diethyl ether, and finally air-dried. Yield: 0.87 g (53%) of a black powder. D.p.: > 250 °C; IR (KBr): $\bar{\nu }$ = 33.82 (OH), 1622 (C=N), 564 (Fe–O), 467 (Fe–N) cm^−1^; UV–Vis (DMF, *c* = 10^−3^ mol dm^−3^): *λ*_max_ = 520, 765 nm.

### Biological studies

In vitro antibacterial activity studies were carried out using the standardized disk-agar diffusion method [74] to investigate the inhibitory effect of the synthesized ligand and its complexes against Gram-positive bacteria, such as *S. aureus* (ATCC25923), Gram-negative bacteria, such as *P. fluorescence* (S97), and *F. oxysporum* as a fungus. The antibiotic chloramphenicol was used as a standard reference in the case of Gram-negative bacteria, cephalothin for Gram-positive bacteria, and cycloheximide as an antifungal standard reference.

The tested compounds were dissolved in DMF, which does not have an inhibition activity, to get concentrations of 2 and 1 mg cm^−3^. The test was performed on potato dextrose agar (PDA) as a medium, which contains an infusion of 200 g potatoes, 6 g dextrose, and 15 g agar [75, 76]. Uniform size filter paper disks (3 disks per compound) were impregnated by an equal volume of the specific concentrations of the dissolved compounds for the test and carefully placed on the incubated agar surface. After an incubation for 36 h at 27 °C in the case of bacteria and for 48 h at 24 °C in the case of fungi, the inhibition of the organisms, which was evidenced by clear zone surround, was measured and used to calculate its mean of inhibition zones. The inhibition zone diameter displayed that the tested ligand and its complexes are active against the used bacteria and fungus.

## Electronic supplementary material

Below is the link to the electronic supplementary material.

Supplementary material 1 (DOCX 3880 kb)
