# Supplementary material for: Syntheses, characterization, and biological activity of novel mono- and binuclear transition metal complexes with a hydrazone Schiff base derived from a coumarin derivative and oxalyldihydrazine
Source: Monatsh Chem. 2017 Nov 27;149(2):431–43. doi: 10.1007/s00706-017-2075-9 (PMC5818636; doi:10.1007/s00706-017-2075-9)
Supplement: Supplementary file 1 — Supplementary material 1 (DOCX 3880 kb) [file 706_2017_2075_MOESM1_ESM.docx]

Syntheses, characterization and biological activity of novel mono and binuclear transition metal complexes with a hydrazone Schiff base derived from a coumarine derivative and oxalyldihydrazine

**Esther Theresa Knittl^1^ ● Azza. A. Abou-Hussein^2^ ● Wolfgang Linert^1^**

^1^Institute of Applied Synthetic Chemistry, Vienna University of Technology, Getreidemarkt, 9/163-AC, 1060 Vienna, Austria.

^2^Faculty of Women for Arts, Science and Education, Ain Shams University, Heliopolis, Cairo, Egypt.

**SupplementaryMaterials**

Index:

S1. Mass spectrum of Schiff base ligand

S2. ^1^H- and ^13^C-NMR of the Schiff base in DMSO

S3. IR spectra of the Schiff base and metal complexes.

S4. Mass spectrum of [Co(H_3_L)(NO_3_)]·2H_2_O (1)

S5. Mass spectrum of Co_2_(H_2_L)(NO_3_)_2_(H_2_O)_2_ (6)

S6. Thermal decomposition of complexes (4), (5) and (10)


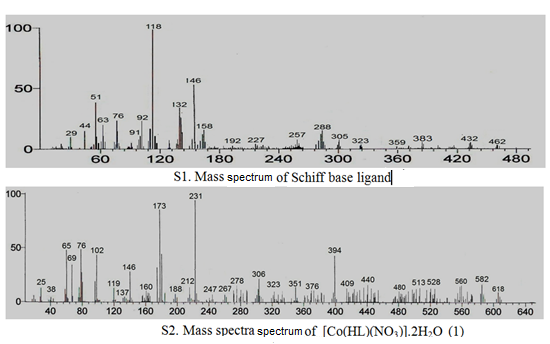

**S1**. Mass spectrum of H_4_L and schematic fragmentation


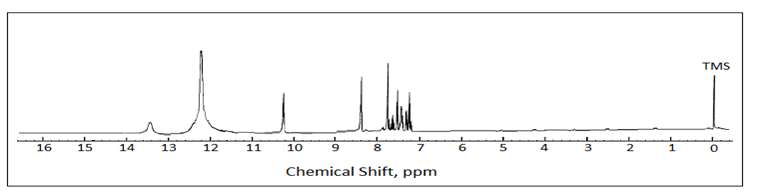


**S2a.** ^1^H-NMR chemical shifts (δ, ppm) of the Schiff base in DMSO


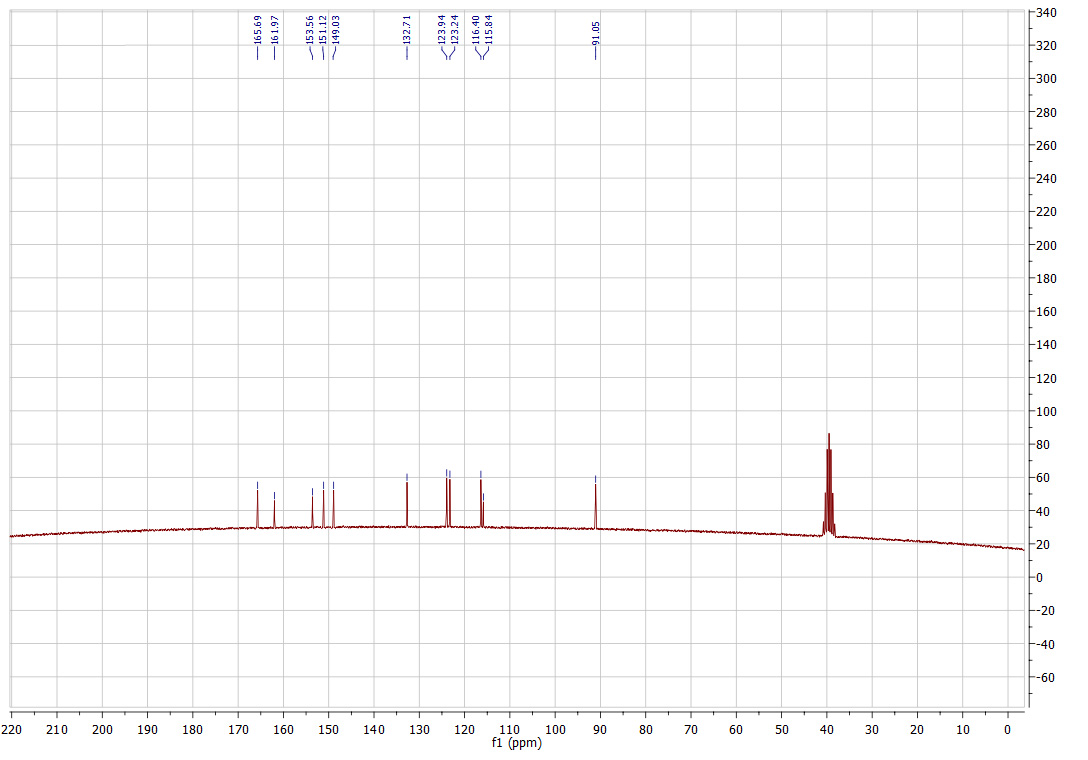


**S2b.** ^13^C-NMR chemical shifts (δ, ppm) of the Schiff base in DMSO

S3. IR spectra of the Schiff base and metal complexes.


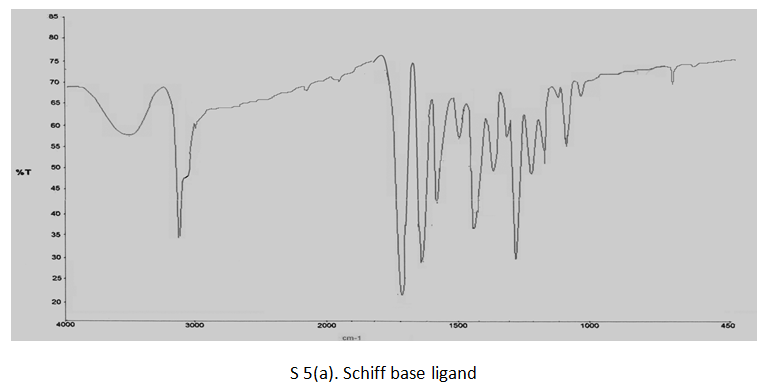


S3 (a) IR spectrum of H_4_L


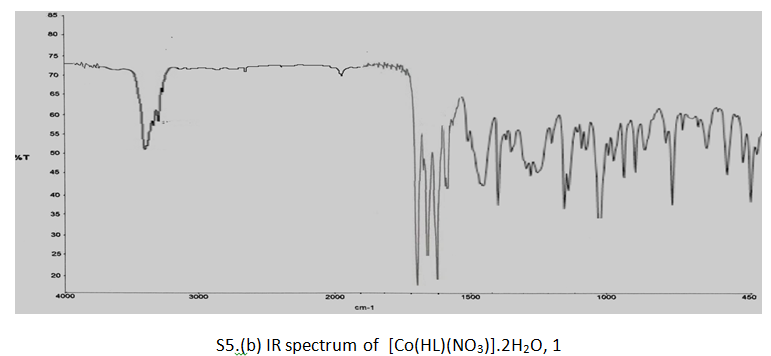
 S3 (b). IR spectrum of (1)


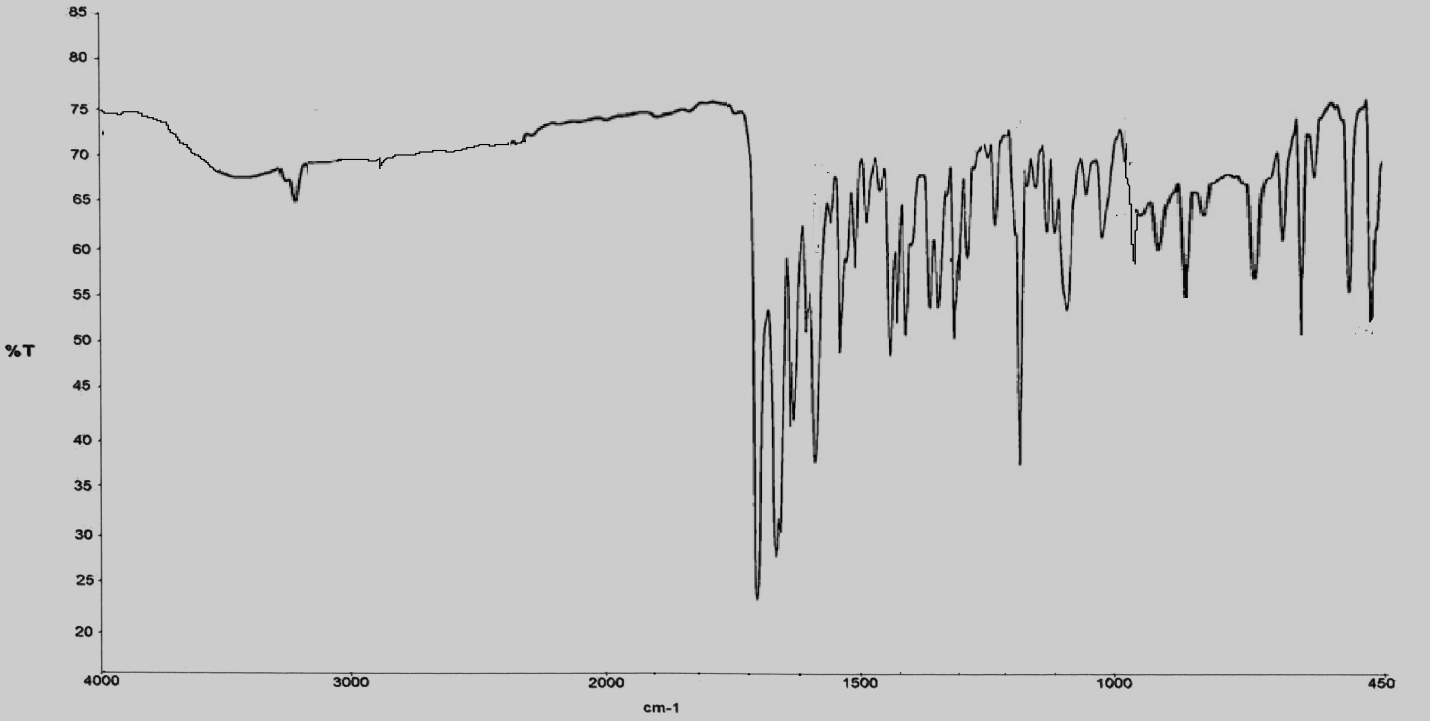


S3 (c). IR spectrum of (2)


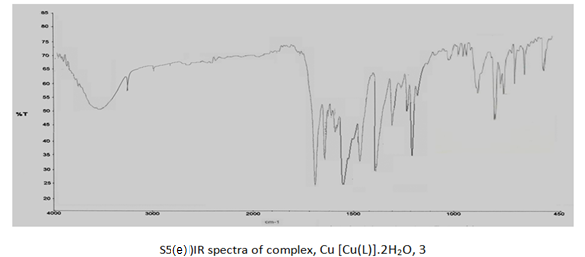


S3 (d). IR spectrum of (3)


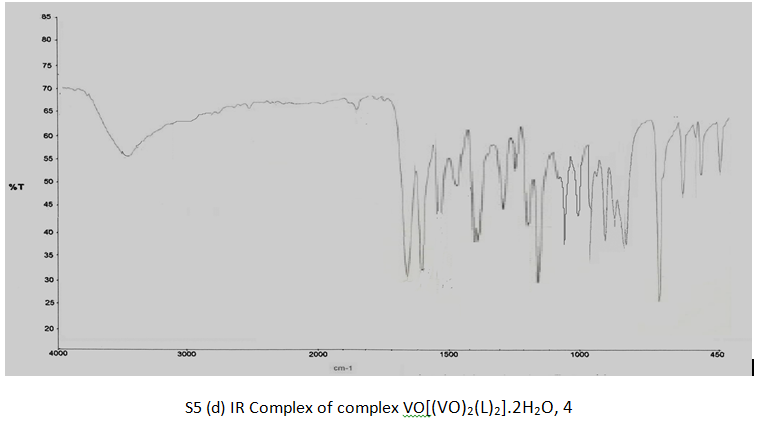


S3 (e). IR spectrum of (4)


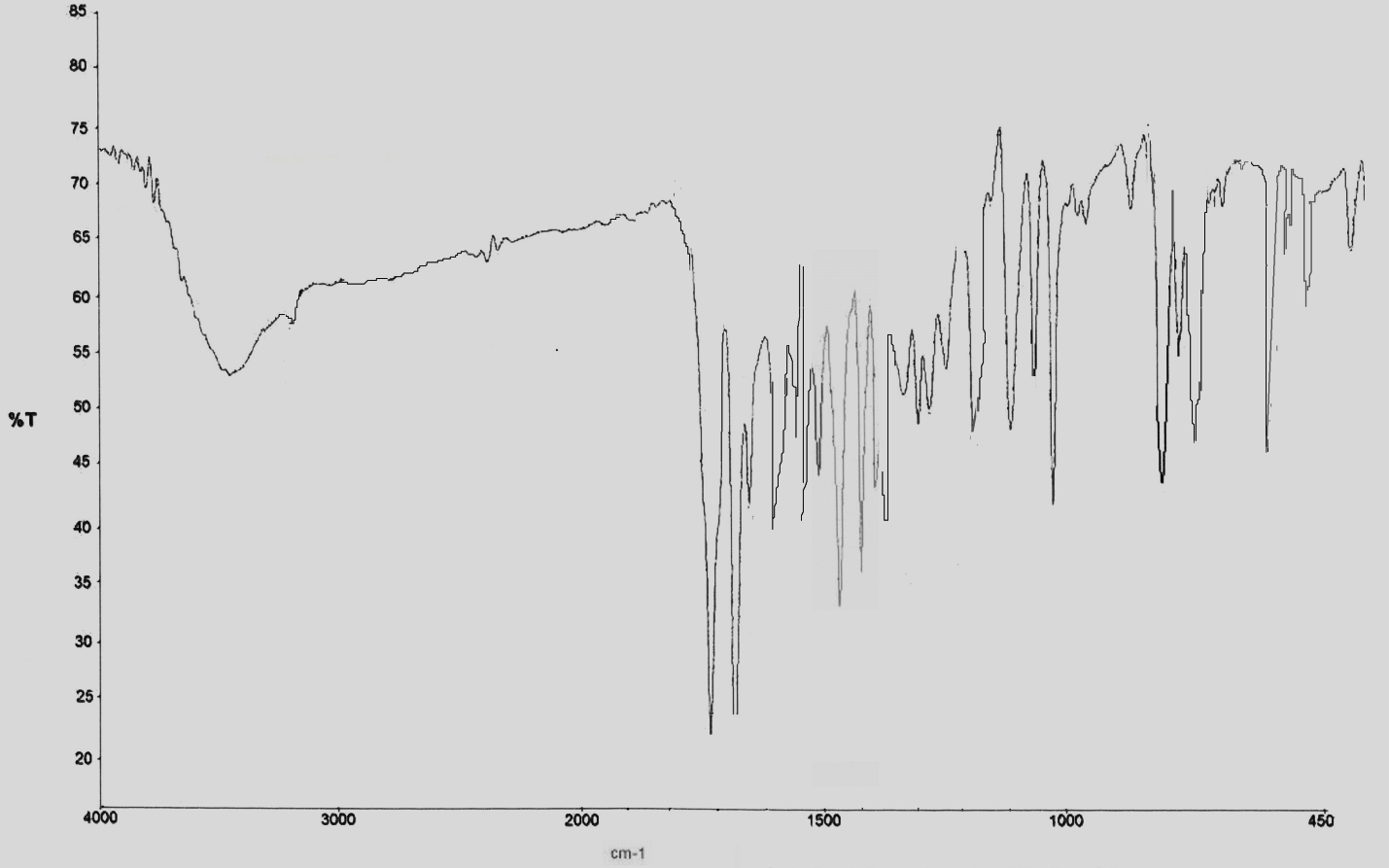


S3 (f). IR spectrum of (5)


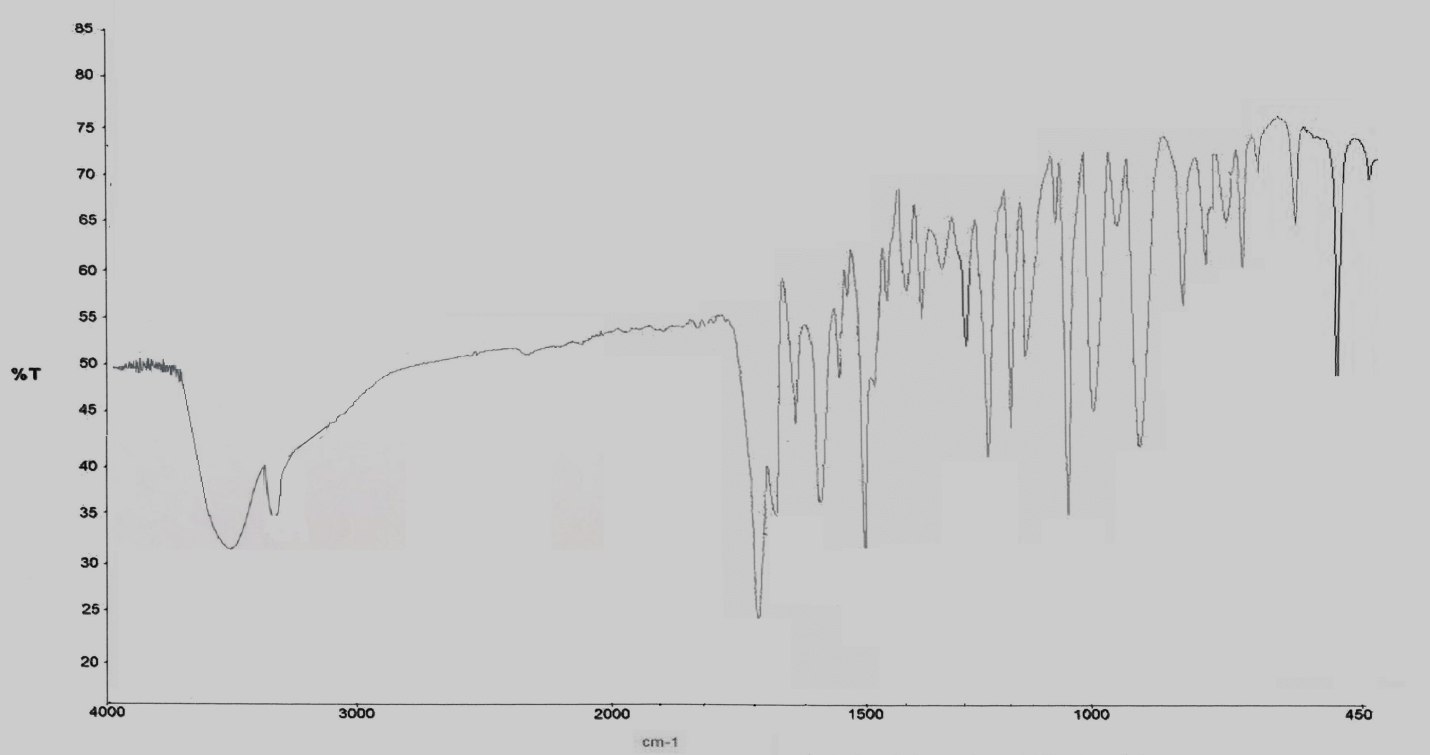


S3 (g). IR spectrum of (6)


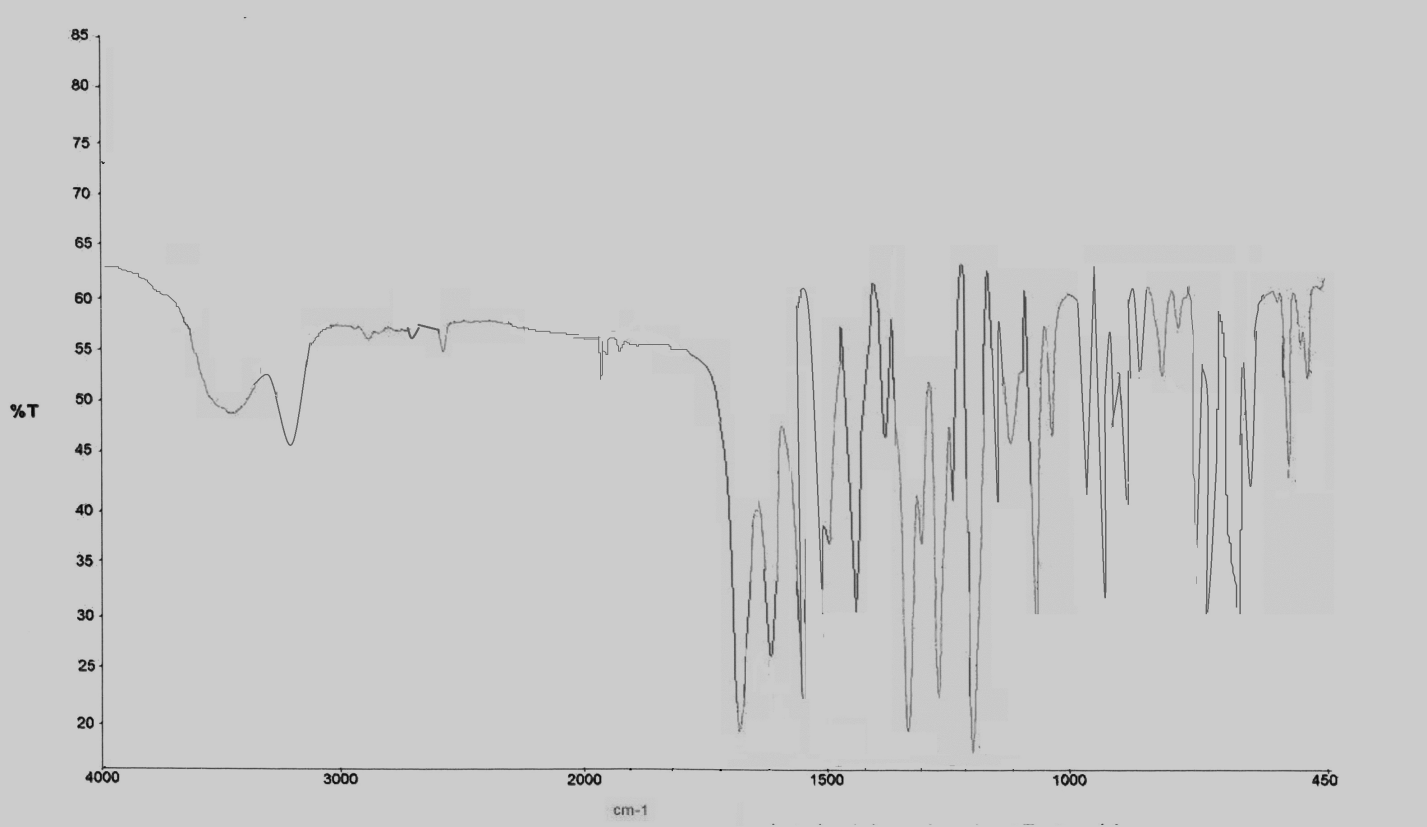


S3 (h). IR spectrum of (7)


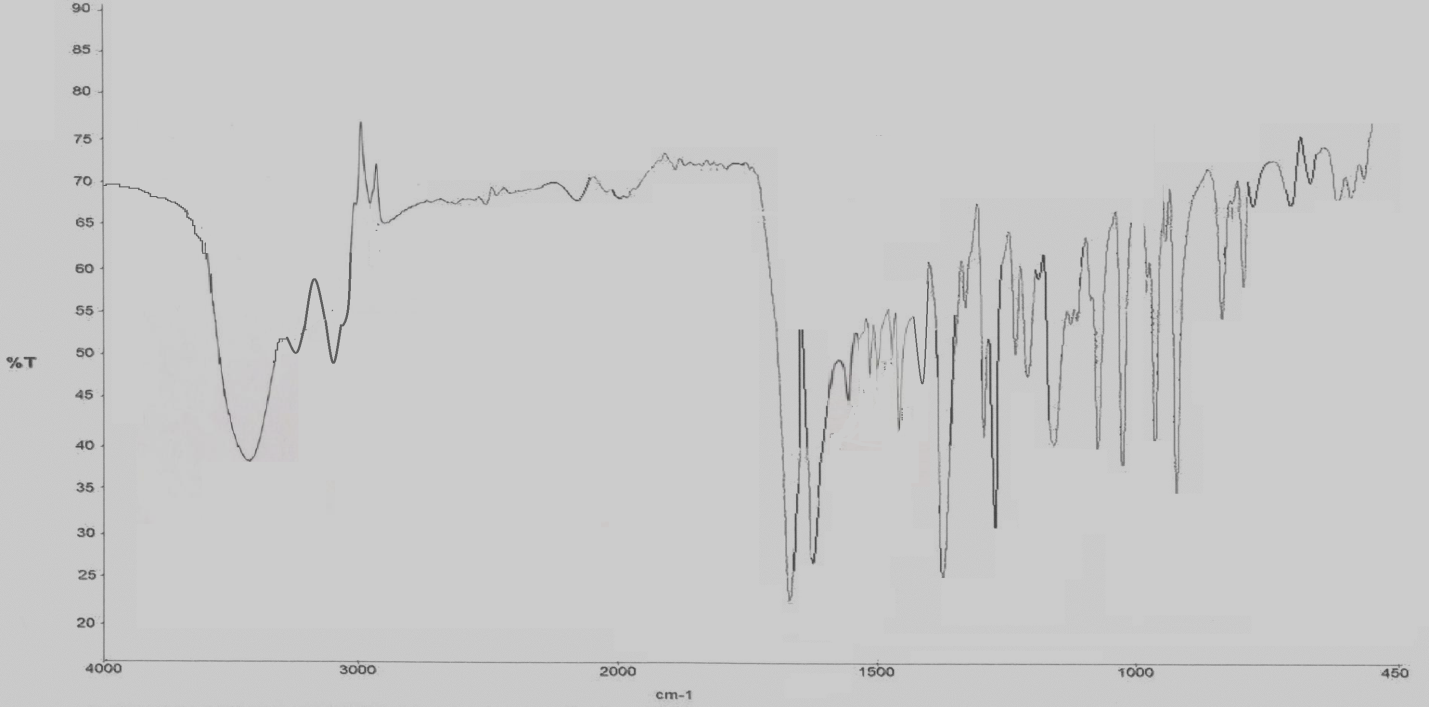


S3 (i). IR spectrum of (8)


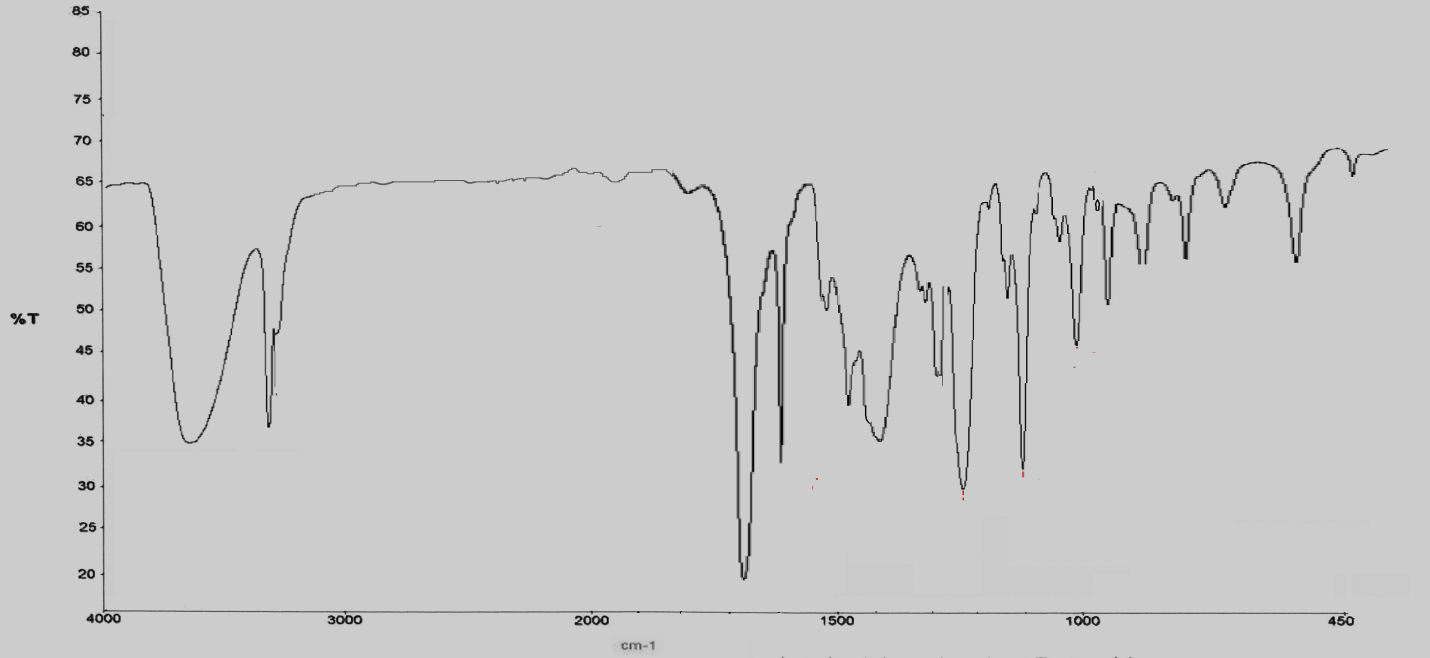


S3 (j). IR spectrum of (9)


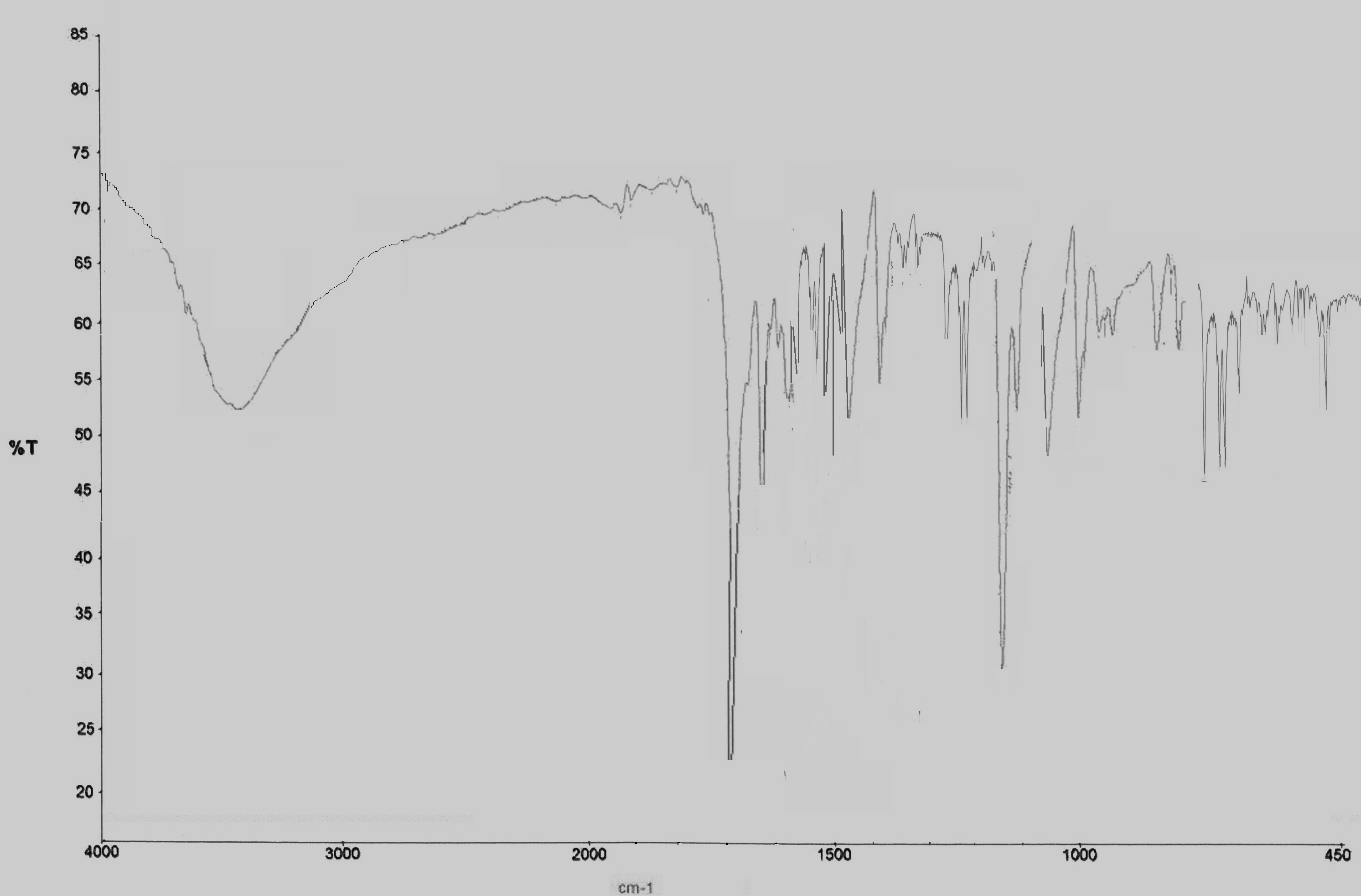


S3 (k). IR spectrum of (10)


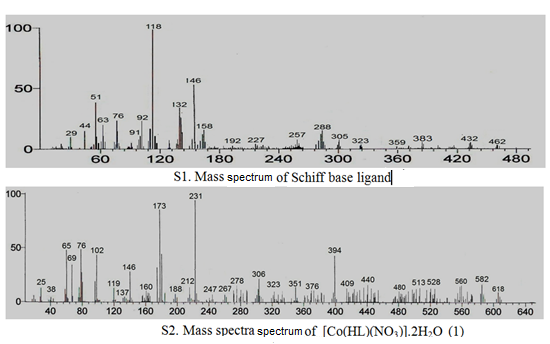

**S4**. Mass spectrum and schematic fragmentation of [Co(H_3_L)(NO_3_)]·2H_2_O (1)


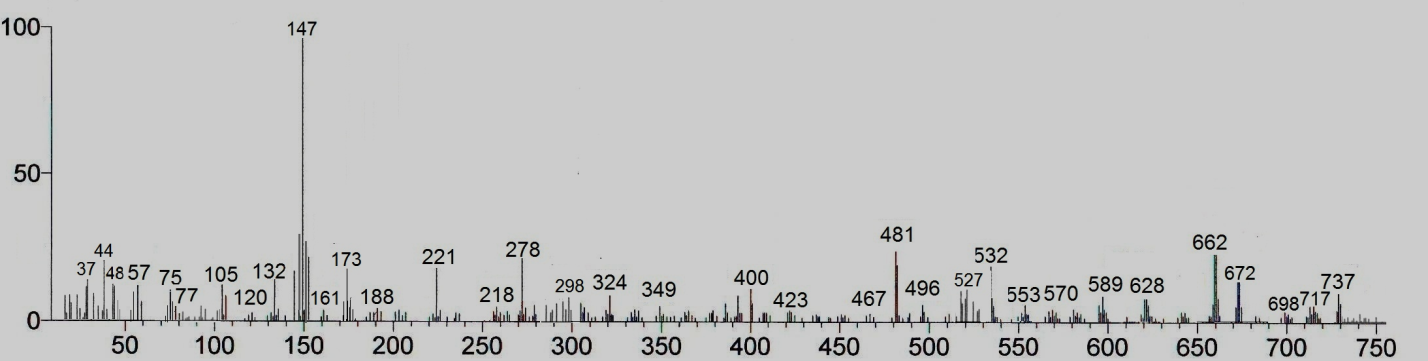

**S5**. Mass spectrum of Co_2_(H_2_L)(NO_3_)_2_(H_2_O)_2_ (6)

S6. Thermal decomposition of complexes (4), (5) and (10)


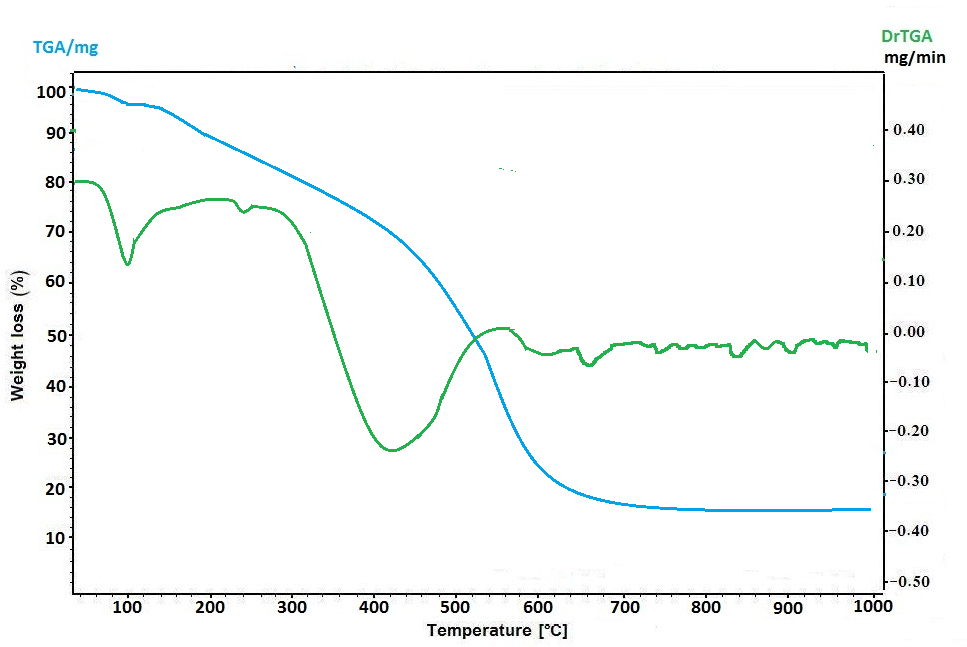


**S6 (a):** TGA/DrTGA curve of **(4)**


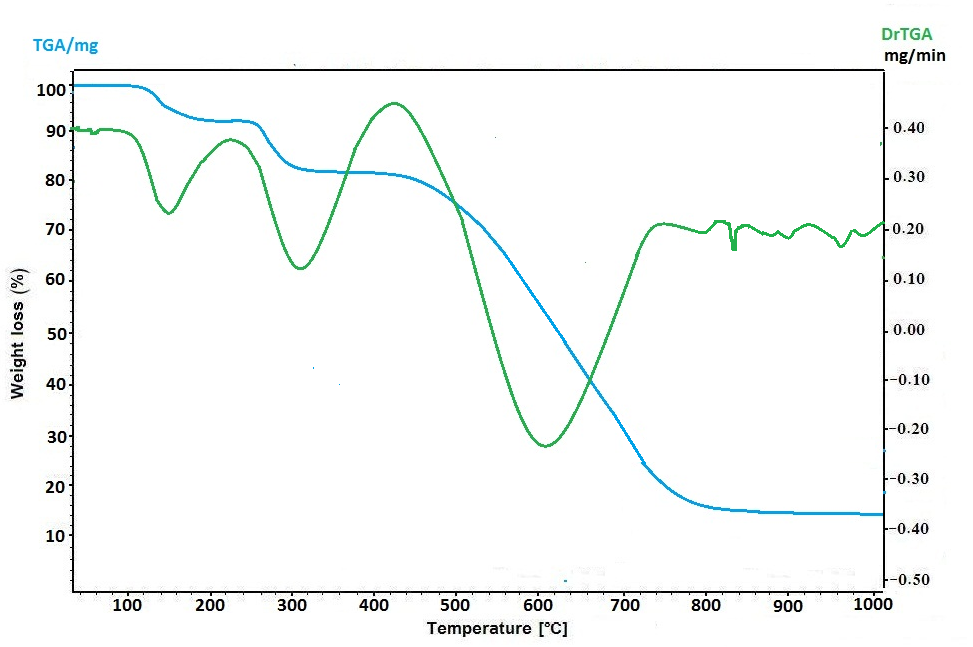


**S6 (b):** TGA/DrTGA curve of **(5)**


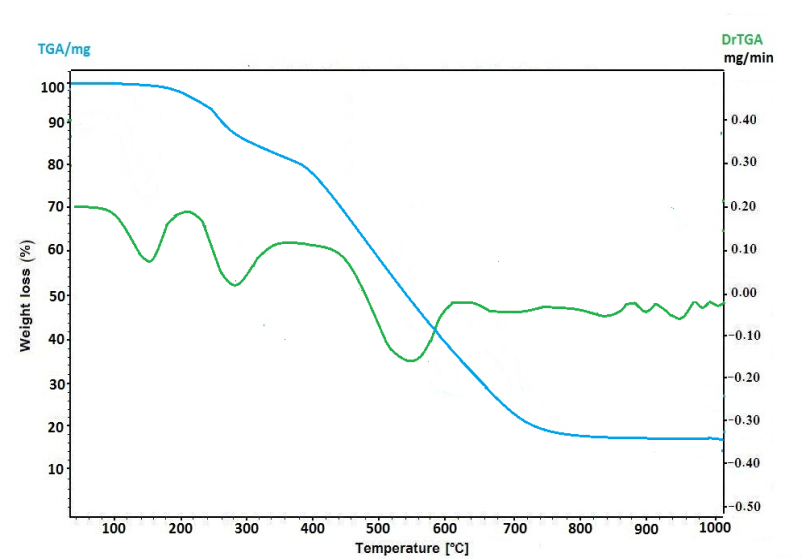


**S6 (c):** TGA/DrTGA curve of **(10)**

**S6 (d):** Schematic fragmentation pathway for [(VO)_2_(H_2_L)_2_]·2H_2_O **(4)**

**S6 (e):** Schematic fragmentation pathway for Fe_2_(H_2_L)_2_(NO_3_)_2_(H_2_O)_2_ **(5)**

**S6 (f):** Schematic fragmentation pathway for Fe_2_(L)(NO_3_)_2_(H_2_O)_4_ **(10)**
